# Controlled Right Ventricular Pressure Overload Can Rescue Left Ventricular Dysfunction by Promoting Biventricular Adaptive Hypertrophy

**DOI:** 10.1016/j.jacbts.2025.02.018

**Published:** 2025-06-18

**Authors:** Matteo Ponzoni, Azadeh Yeganeh, Libo Zhang, Jennifer Zhang, Ramesh B. Vanama, Kaley Hogarth, Quynh N. Phi, Jing Li, Loukmane Karim, John G. Coles, Jason T. Maynes

**Affiliations:** a Division of Cardiovascular Surgery, The Hospital for Sick Children, Toronto, Ontario, Canada; b Program in Molecular Medicine, SickKids Research Institute, Toronto, Ontario, Canada; c Department of Anesthesia and Pain Medicine, The Hospital for Sick Children, Toronto, Ontario, Canada; d Department of Anesthesiology and Pain Medicine, University of Toronto, Toronto, Ontario, Canada

**Keywords:** dilated cardiomyopathy, experimental model, left ventricular dysfunction, pulmonary artery banding

## Abstract

Pulmonary artery banding (PAB) has been investigated as a therapy for left ventricular (LV) dysfunction in pediatric dilated cardiomyopathy; however, the underlying mechanisms of action of PAB-induced LV rehabilitation remain unknown. This study aims to establish a small animal model of PAB-treated LV dysfunction to document the biventricular hemodynamic and tissue-level modifications promoted by PAB. Sprague-Dawley rats underwent left anterior descending (LAD) artery ligation (LV dysfunction model) followed by PAB 1-week post-injury (LAD + PAB, n = 13). Controls consisted of sham (n = 16), PAB-only (n = 16), and LAD rats (n = 15). The animals underwent weekly echocardiography and terminal histopathology 4 weeks after surgery. Data shown as mean ± SEM or median (Q1-Q3). LAD + PAB rats exhibited positive LV remodeling (LV end-diastolic volume: 0.49 ± 0.02 mL vs 0.66 ± 0.03 mL; *P* < 0.001), improvement of LV ejection fraction (0.48 ± 0.01 vs. 0.36 ± 0.01; *P* < 0.001), and normalization of mitral valve Doppler E/A (1.43 ± 0.03 vs 1.91 ± 0.09; *P* < 0.001) compared to LAD animals. Histologic analysis documented LV hypertrophy (wall thickness/body weight: 9.3 ± 0.4 μm/g vs 7.2 ± 0.3 μm/g; *P* = 0.005), increased LV cardiomyocyte diameter (14.8 [Q1-Q3: 13.9-15.7] μm vs 11.3 [Q1-Q3: 10.8-11.7] μm; *P* = 0.001), and augmented neoangiogenesis (6.5 ± 0.2 vessels/mm^2^ vs 4.7 ± 0.5 vessels/mm^2^; *P* = 0.005) in LAD + PAB vs LAD hearts. Mechanistically, we observed reduced LV fibrosis (9.8% [Q1-Q3: 7.7%-13.4%] vs 17.4% [Q1-Q3: 14.8%-20.2%]; *P* = 0.003) and fibroblast cellular senescence (5.7% [Q1-Q3: 4%-10.7%] vs 16.1% [7.6%-18.4%], *P* = 0.029), and preserved phospholamban (PLN) phosphorylation in the LV of LAD + PAB vs LAD rats (increased PLN/PLN: 0.6 ± 0.1 vs 1.0 ± 0.1; *P* = 0.008). In our model, PAB induced positive LV remodeling and improved LV systolic-diastolic function. PAB stimulated biventricular compensated hypertrophy that may constitute a potential adaptive mechanism which can rescue residual LV function and limit LV fibrosis/injury.

## Highlights

• In our new rodent model of LV dysfunction treated with PAB, right ventricular pressure overload reduces LV dilatation and improves LV systolic and diastolic function.
• PAB promotes biventricular hypertrophy and neoangiogenesis, reduces LV fibrosis and cellular senescence, and preserves calcium handling pathways.
• PAB-induced LV improvements are supported by reduced phospholamban dephosphorylation, increased angiogenesis, and normalized prohypertrophic markers across both ventricles.
• Our research supports PAB as a promising bridge-to-recovery therapy in pediatric LV failure by recruiting residual LV function and limiting LV injury.

## Visual Abstract

**Figure:**
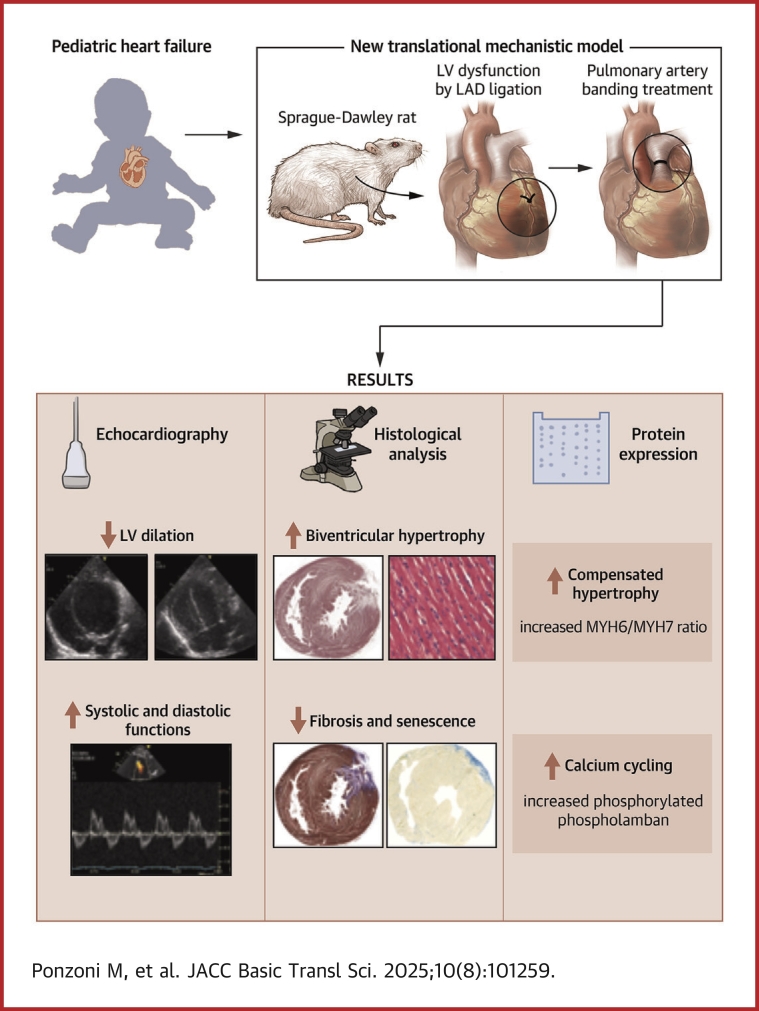

Dilated cardiomyopathy (DCM) is the leading cause of pediatric end-stage heart failure,^1^^,^^2^ with less than half of affected children reaching adulthood. Surgical therapies include heart transplantation and mechanical circulatory support, each with significant morbidities,^3^^,^^4^ especially in smaller infants. Pulmonary artery banding (PAB) has recently been re-investigated as a successful bridge-to-recovery strategy for infants with DCM.^5^, ^6^, ^7^, ^8^ Clinical experience has shown that a controlled right ventricular (RV) pressure overload can acutely induce a leftward shift of the interventricular septum, which constrains left ventricular (LV) dilatation in the context of DCM, and ameliorates LV systolic and diastolic dysfunction.^5^^,^^9^, ^10^, ^11^

In addition to the immediate hemodynamic benefits created by the positive ventricular-ventricular interactions, a sustained response has been observed in children with DCM treated with PAB, with long-term LV functional recovery occurring in as high as 78% of treated patients.^10^ These results suggest a possible activation of unelucidated compensatory and/or proadaptive molecular and cellular pathways in the myocardium.^7^ However, heterogeneity in clinical results, lack of appropriate selection criteria, and under-use of this approach in DCM persist as a result of an incomplete understanding of the mechanisms which mediate PAB-induced LV functional recovery.

We describe a small animal model of LV dysfunction treated with PAB, facilitating observation and quantification of the acute and chronic LV hemodynamic changes in response to RV pressure overload, as well as the tissue-level and cellular modifications that occur in the myocardium of both ventricles. Controlled PAB improved LV systolic and diastolic function and induced a biventricular compensated hypertrophic phenotype and induced recruitment of LV functional reserve. These data provide new insights into myocardial adaptation/retraining potential and reveal beneficial cellular effects of ventricular-ventricular interactions resulting from PAB, potentially helping to clarify patient selection, timing of intervention, and expectations for recovery without further surgical intervention.

## Methods

### Experimental protocol

**Figure 1:**
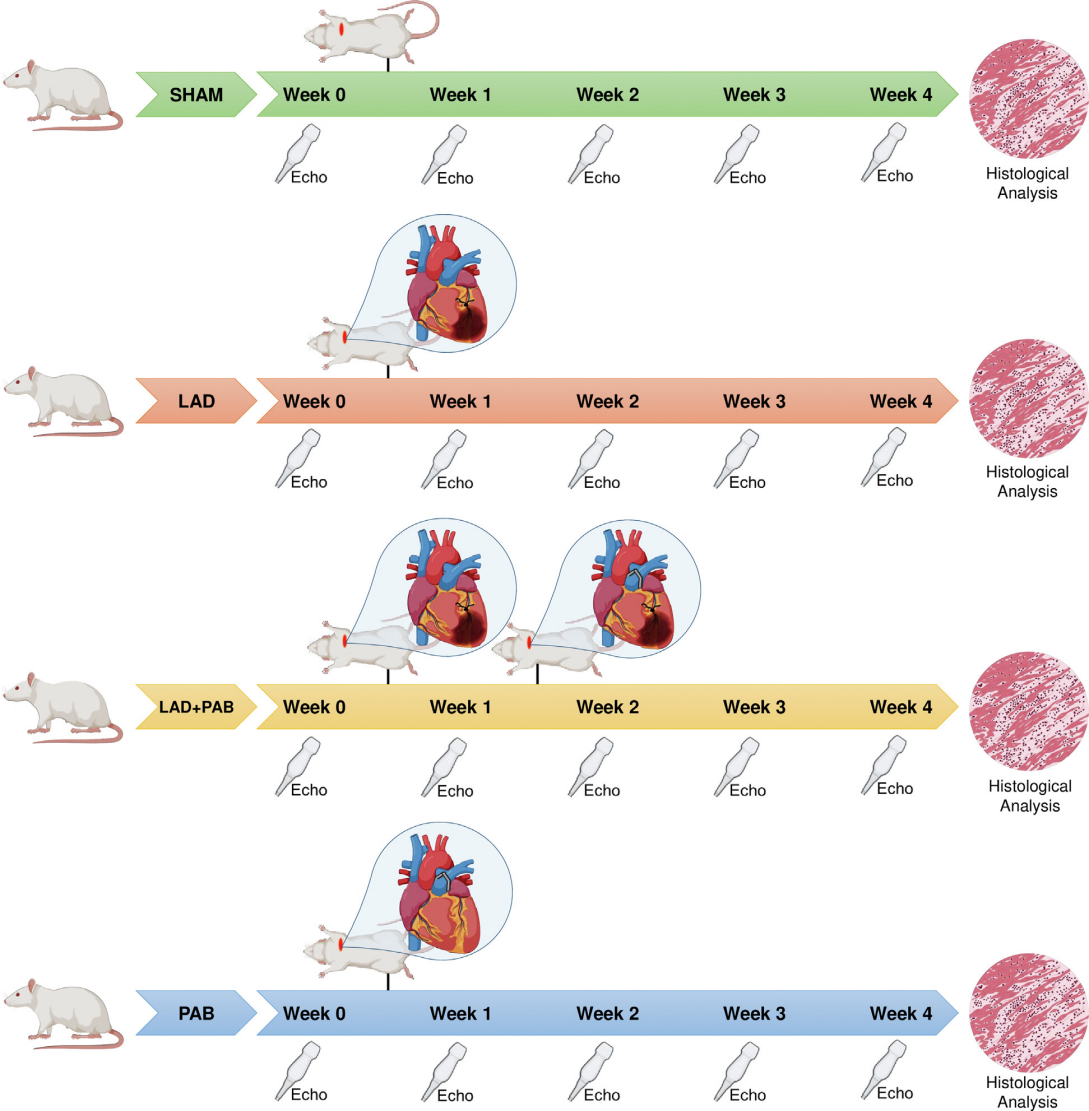
Experimental Protocol Rats were assigned to sham, left anterior descending artery (LAD), LAD + pulmonary artery banding (PAB), and PAB surgery and followed with weekly echocardiography (Echo) until the experiment termination (week 4).

Six- to 8-week-old Sprague-Dawley rats (Charles River), equally distributed between males and females, were randomly assigned to the following groups: left anterior descending (LAD) artery ligation, PAB, LAD ligation followed by PAB 1 week post-injury (LV dysfunction rescued by PAB model), and sham surgery. Animals underwent weekly echocardiography and terminal histopathology after 4 weeks. Total tissue LV and RV lysates were processed for western blot analysis. Detailed methods are described in the Supplemental Appendix. The experimental protocol (Figure 1) was approved by the SickKids Animal Care Committee (study #64412) and performed in accordance with national and institutional guidelines.

### Statistical analysis

Normality of data was checked with the Shapiro-Wilk test. Data are presented as mean ± standard error mean or median with 25th-75th percentiles (Q1-Q3), dependent on data distribution. Data with more than 2 groups were compared using one-way analysis of variance with Tukey's post hoc test or the Kruskal-Wallis test with Dunn's post hoc test for multiple comparisons, and data with 2 groups were compared using an unpaired Student’s *t*-test or Mann-Whitney test, as appropriate. Statistical significance was set at *P* < 0.05. Analyses were performed using SPSS 23.0 (IBM Corporation) and GraphPad Prism 9.5.1 (GraphPad Software).

## Results

### Echocardiographic results

**Figure 2:**
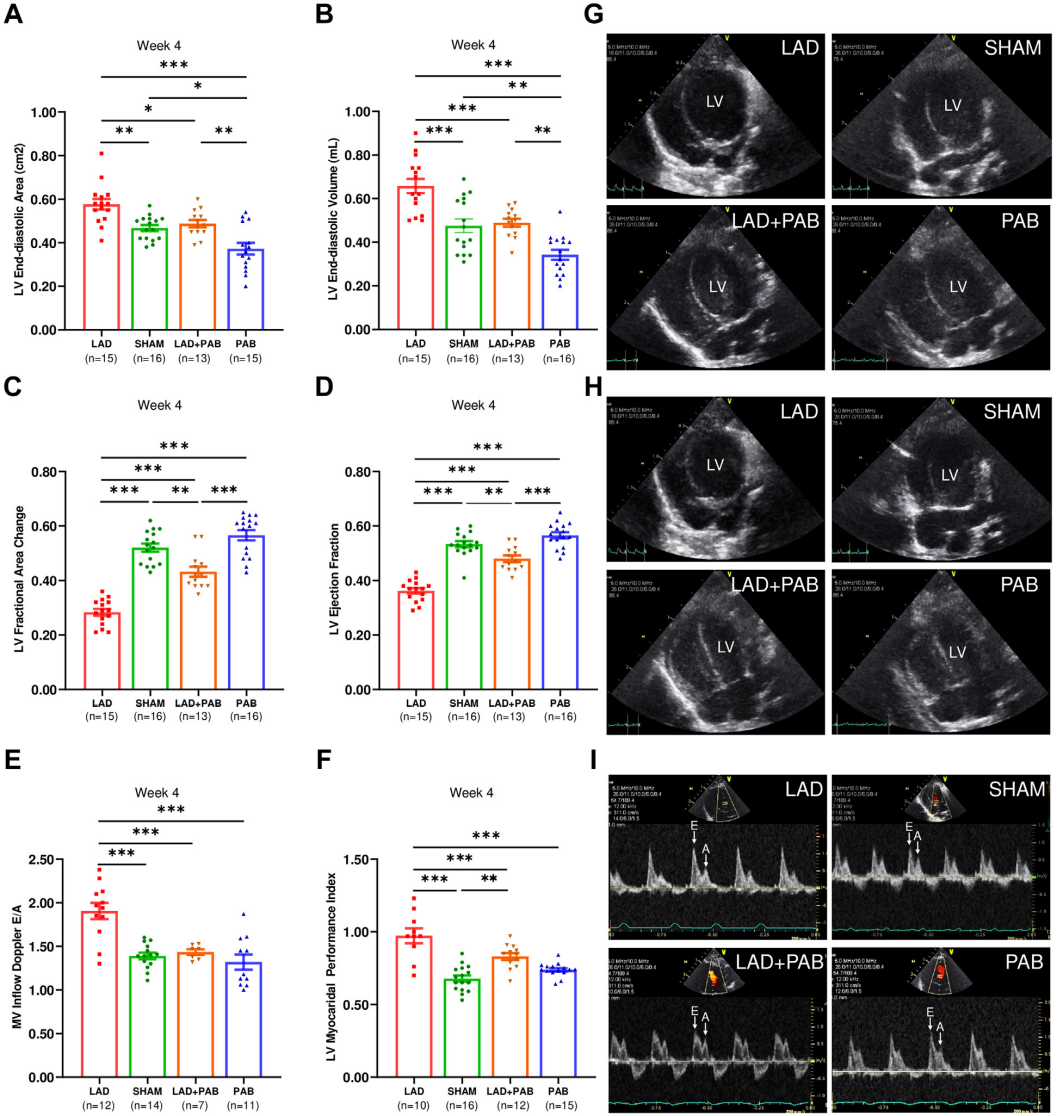
PAB Reduces LV Dilatation and Improves LV Systolic and Diastolic Function In LAD + PAB animals, left ventricular (LV) end-diastolic area (A) and end-diastolic volume (B) were reduced compared to LAD rats. LV fractional area change (C) and LV ejection fraction (D) improved in LAD + PAB vs LAD rats. LV diastolic function was improved in LAD + PAB vs LAD animals, as measured by reduced mitral valve (MV) inflow Doppler E/A (E) and LV myocardial performance index (F). (G,H) Echocardiographic 4-chamber views during end-diastolic (G) and end-systolic (H) phases. There is LV dilatation in LAD, which is reduced in LAD + PAB due to the leftward shift of the intraventricular septum. (I) Examples of MV inflow Doppler waves. Data are presented as mean ± standard error of mean and compared using one-way analysis of variance with Tukey post hoc test (A-F). ∗*P* < 0.05. ∗∗*P* < 0.01. ∗∗∗*P* < 0.001. Other abbreviations as in Figure 1.

A total of 16 rats in sham, 16 in PAB, 15 in LAD, and 13 in LAD + PAB groups survived until experiment termination. Early after arterial ligation (week 1), LAD and LAD + PAB rats displayed LV dilatation, impaired LV contractility, and deteriorated LV diastolic function compared to sham and PAB groups (Supplemental Figure 1). With PAB treatment, LV dilatation was reduced in LAD + PAB compared to LAD animals; in the latter, LV chamber enlargement persisted until the experiment termination (Figures 2A and 2B, Supplemental Figure 2). LV fractional area change and ejection fraction substantially improved with PAB in the LAD + PAB group, whereas, in the LAD group, LV contractile function remained impaired (Figures 2C and 2D, Supplemental Figure 2). Similarly, mitral valve inflow Doppler E/A and LV myocardial performance index was reduced after PAB treatment in the LAD + PAB group compared to LAD animals (Figures 2E and 2F), suggesting combined improvement in LV diastolic and systolic performance. At week 4, RV dilatation was observed in LAD + PAB and PAB groups, with a small impairment of RV contractile function (Supplemental Figure 3). PAB induced significant RV pressure overload (as measured by continuous-wave Doppler across the PAB), RV hypertrophy, and LV posterior wall hypertrophy, which occurred in the absence of LV outflow tract obstruction (Supplemental Figures 3 and 4). With all metrics, we did not observe any sex-specific differences between the groups. These findings suggest a beneficial effect of PAB in reducing pathological LV dilatation after an ischemic insult and ameliorating LV systolic and diastolic dysfunction consistent with the induction of a compensated hypertrophic phenotype in the LV.

### Macroscopic examination

**Figure 3:**
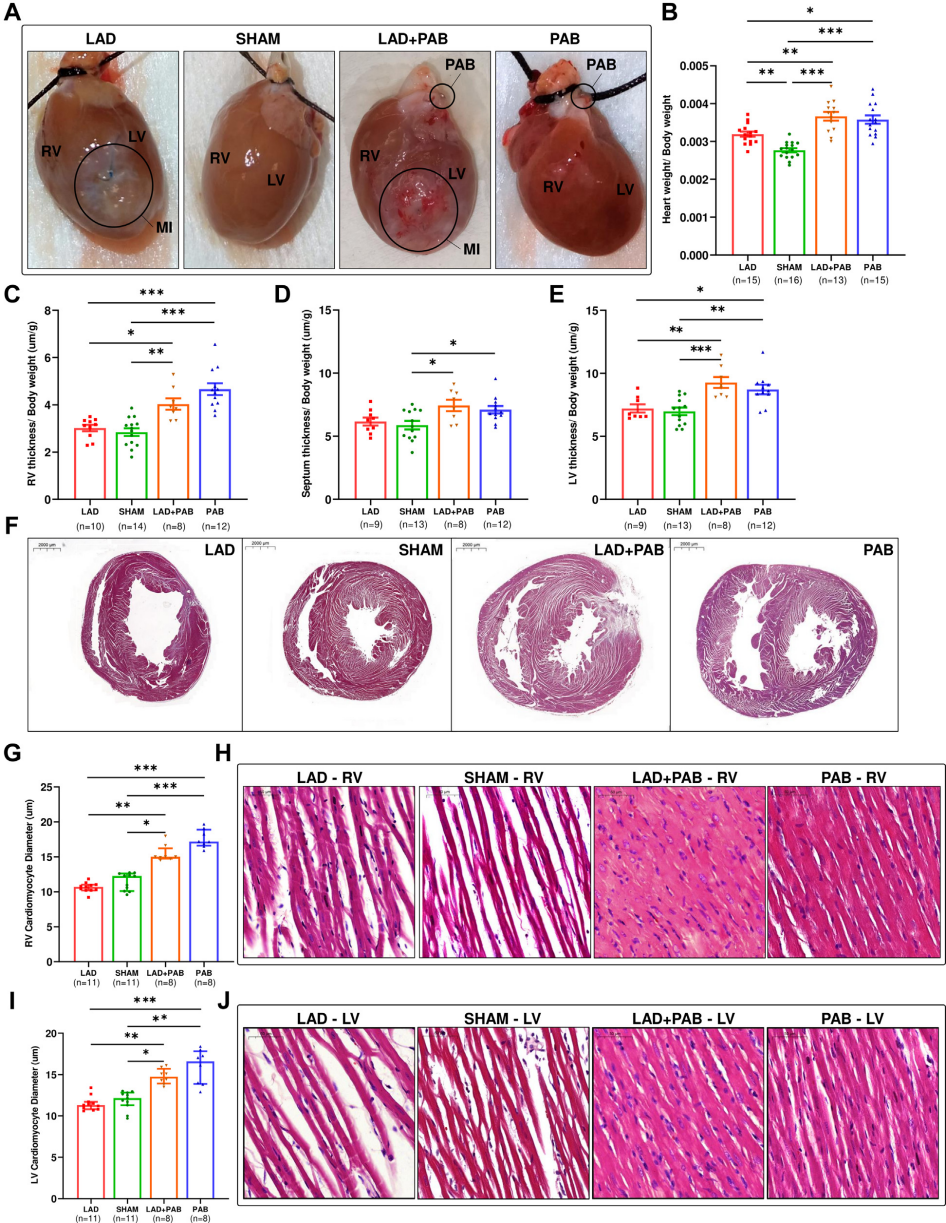
PAB Induces Hypertrophy and Increased Cardiomyocyte Diameter (A) Hearts showing LV myocardial infarction (MI) scar in LAD and LAD + PAB, and the PAB clip in LAD + PAB and PAB. Total heart weight was higher in LAD + PAB and PAB groups compared to LAD (B). Right ventricular (RV) thickness was increased in the LAD + PAB and PAB groups compared to LAD and sham (C). Interventricular septum thickness was higher in LAD + PAB and PAB compared to sham (D). LV posterior wall thickness was increased in LAD + PAB and PAB compared to LAD and sham (E). (F) Examples of hematoxylin and eosin staining highlighting biventricular hypertrophy in LAD + PAB and PAB hearts. Cardiomyocyte diameter was increased in PAB and LAD + PAB compared to LAD and sham (RV free wall [G, H]; LV posterior and lateral wall [I, J]). Data are presented as mean ± standard error of mean and compared using one-way analysis of variance with Tukey post hoc test (B-E) or as median (Q1-Q3) and compared with Kruskal-Wallis test with Dunn's post hoc test (G, I). ∗*P* < 0.05. ∗∗*P* < 0.01. ∗∗∗*P* < 0.001. Abbreviations as in Figures 1 and 2.

Macroscopic examination of the heart tissue confirmed the presence of an LV myocardial infarction (MI) scar in LAD and LAD + PAB animals and the correct positioning of the PAB clip in LAD + PAB and PAB animals (Figure 3A). Total heart weight was increased in LAD, LAD + PAB, and PAB groups compared to shams; however, LAD + PAB and PAB groups displayed significantly higher heart weight, suggesting the presence of pressure overload–dependent hypertrophy (Figure 3B).

### Histologic results

At histologic examination, RV free-wall hypertrophy was observed in the LAD + PAB and PAB groups compared to LAD and sham (Figure 3C). Interestingly, LAD + PAB and PAB animals showed increased interventricular septum thickness compared to shams (Figure 3D), as well as LV posterior wall hypertrophy compared to LAD and sham animals (Figures 3E and 3F), paralleling the echocardiographic findings. To confirm the presence of cellular hypertrophy causing increased biventricular thicknesses, we measured cardiomyocyte diameter. LAD + PAB and PAB groups displayed increased cardiomyocyte cross-sectional diameter compared to LAD and sham animals in the RV free wall (Figures 3G and 3H) and the LV posterior-lateral wall (Figures 3I and 3J), revealing that the cellular hypertrophic response is not limited to the pressure overloaded RV but also involves the LV.

**Figure 4:**
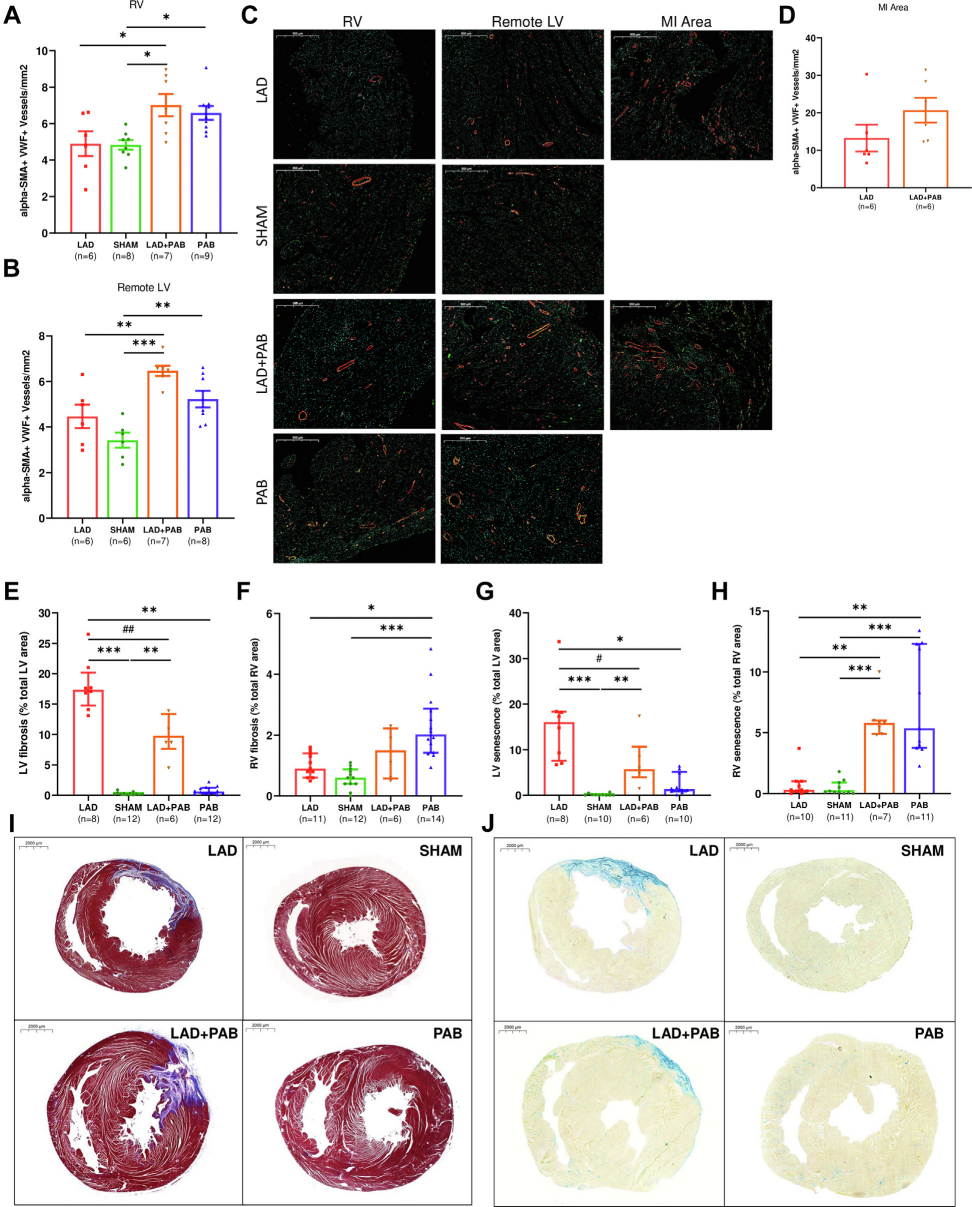
PAB Reduces LV Fibrosis and Cellular Senescence and Promotes Angiogenesis Masson’s trichrome staining shows higher LV fibrosis in LAD vs LAD + PAB hearts (A, E), whereas RV fibrosis was higher in PAB vs sham and LAD hearts (B, E). β-galactosidase activity staining showed higher LV cellular senescence in LAD hearts compared to LAD + PAB (C, F). Conversely, RV cellular senescence was increased in LAD + PAB and PAB groups compared to LAD (D, F). LAD + PAB hearts showed increased vessel density compared to LAD in the RV (G, I) and the remote LV (H) and trended toward higher in the peri-infarct area (L). Data are presented as mean ± standard error of mean and compared using one-way analysis of variance with Tukey post hoc test (A, B) or as median (Q1-Q3) and compared with Kruskal-Wallis test with Dunn's post hoc test (E-H). ∗*P* < 0.05. ∗∗*P* < 0.01. ∗∗∗*P* < 0.001. #*P* < 0.05 and ##*P* < 0.01 by Mann-Whitney test. SMA = smooth muscle actin; VWF = von Willebrand factor; other abbreviations as in Figure 1, Figure 2, Figure 3.

To assess if controlled RV pressure overload can promote a biventricular hypertrophic response with a compensated phenotype, we measured myocardial vessel density using α–smooth muscle actin/von Willebrand factor immunofluorescence staining. In LAD + PAB and PAB hearts, RV vessel density was higher compared to LAD and sham (Figure 4A). Similarly, myocardial vessel density was increased in LAD + PAB and PAB hearts in the remote LV (Figures 4B and 4C), suggestive of a diffuse compensated hypertrophic phenotype. Vessel density in the peri-infarct region trended toward higher in the LAD + PAB group but failed to reach statistical significance (Figure 4D).

To determine if PAB can influence LV fibrotic scarring after acute injury, we performed Masson’s trichrome staining, which showed the presence of significant LV fibrosis in LAD and LAD + PAB animals compared to sham and PAB animals (Figure 4E). However, treatment with PAB reduced the LV fibrotic area (*P* = 0.003, Mann-Whitney). Conversely, RV pressure overload increased the amount of RV fibrosis in PAB animals compared to LAD and sham (Figure 4F), although the total fibrotic area remained a small percentage of the ventricle. LV cellular senescence (β-galactosidase activity staining) was increased in LAD and LAD + PAB hearts compared to sham and PAB groups (Figure 4G); however, the β-galactosidase signal in LAD + PAB was significantly less than that in LAD (*P* = 0.020). Consistent with the ventricular stress applied, PAB induced RV cellular senescence in LAD + PAB and PAB groups compared to sham and LAD (Figure 4H). The areas where cellular senescence was detected directly overlapped the fibrotic lesions in the LV (post-MI scar) and the RV which was mainly focused at RV-LV hinge points (Figures 4I and 4J).

**Figure 5:**
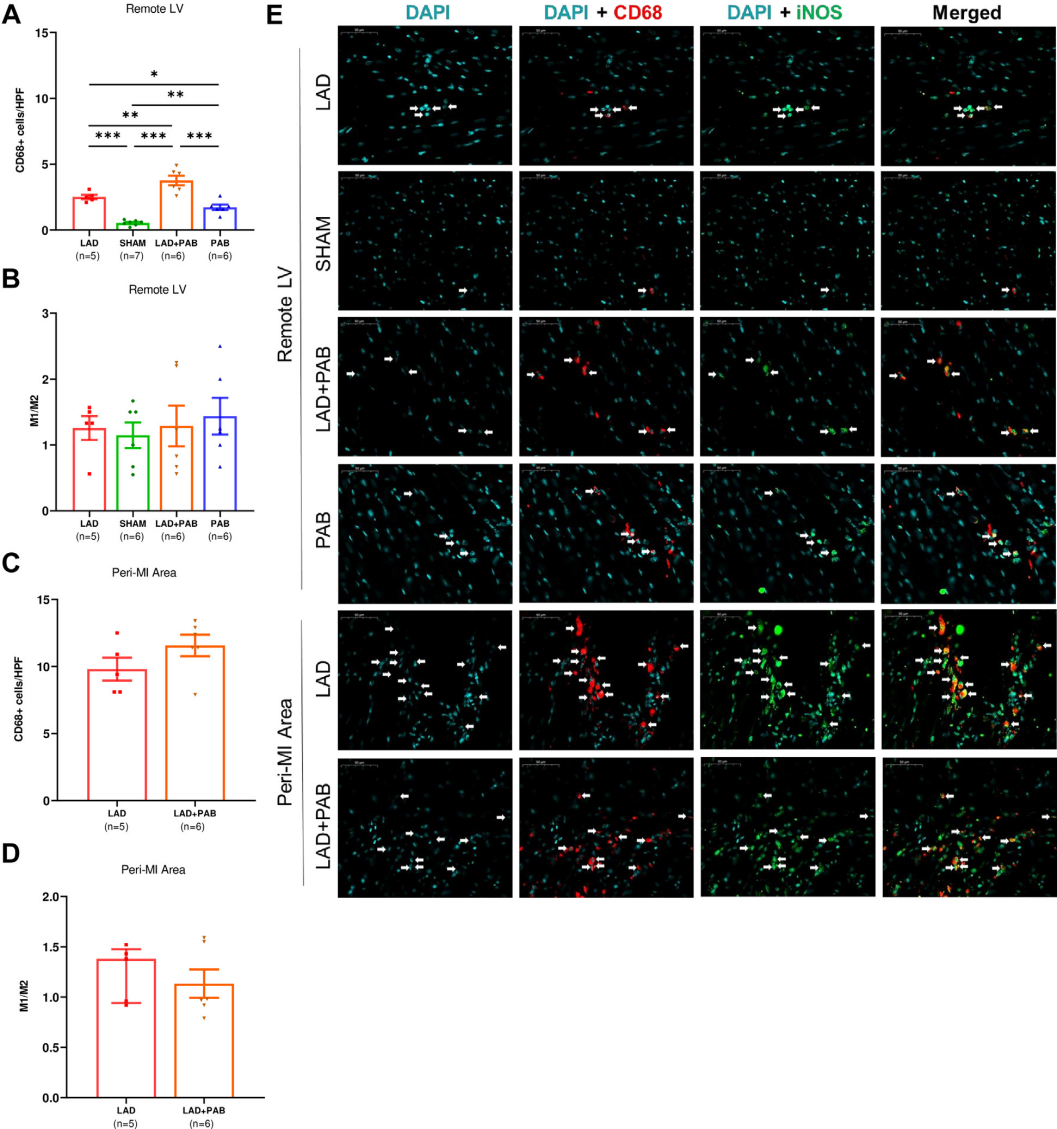
PAB Induces Macrophage Infiltration CD68+ cells were increased in the remote LV of LAD, LAD + PAB, and PAB hearts compared to sham, with overall higher infiltration of macrophages in LAD + PAB hearts (A). No differences in CD68/inducible nitric oxide synthase (iNOS) (M1)/CD68/CD206 (M2) ratio were observed (B). A similar number of macrophages were found in the peri-infarct region in LAD and LAD + PAB hearts (C), with comparable M1/M2 ratio (D). (E) Examples of CD68 (red), iNOS (green), and 4’6-diamidino-2-phenylindole (DAPI) (blue) staining. Data are presented as mean ± standard error of mean and compared using one-way analysis of variance with Tukey post hoc test (A, B) or Student’s *t*-test (C, D). ∗*P* < 0.05. ∗∗*P* < 0.01. ∗∗∗*P* < 0.001. Other abbreviations as in Figure 1, Figure 2, Figure 3.

To assess the potential role of RV pressure overload in modulating the inflammatory cell infiltrate after LV injury, we performed immunofluorescence staining for the macrophage marker CD68, which documented an increased infiltration of macrophages in the remote LV in LAD, LAD + PAB, and PAB hearts compared to sham with the overall highest number of macrophages detected in LAD + PAB hearts (Figure 5A). To investigate macrophage polarization, CD68/inducible nitric oxide synthase (M1) and CD68/CD206 (M2) immunostaining was performed. No differences in M1/M2 polarization were observed among groups in the remote LV (Figure 5B). A nonstatistical difference in the infiltration of macrophages was found in the peri-infarct region in LAD and LAD + PAB hearts (Figure 5C), with similar M1/M2 composition (Figure 5D, Supplemental Figure 5). Conversely, higher CD68+ macrophages infiltration was observed in the RV of LAD + PAB and PAB animals compared to sham (Supplemental Figure 6), with a predominant M1 polarization (Supplemental Figure 6), suggesting a proinflammatory activation of macrophages in response to PAB-related ventricular stress.

### Western blot analysis

**Figure 6:**
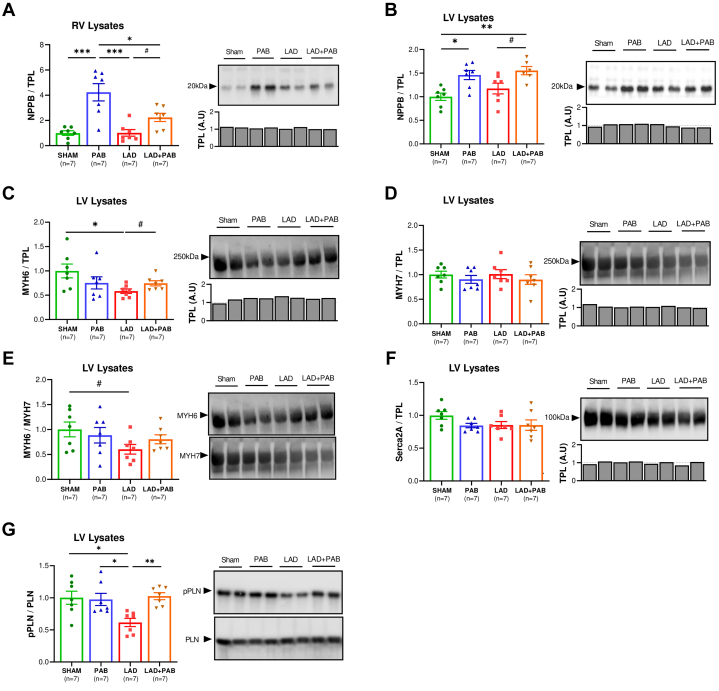
PAB Triggers Biventricular Molecular Hypertrophic Signaling and Preserves Calcium Handling in the Injured LV Western blot analysis of lysates from the RV free wall and LV posterior wall showed increased natriuretic peptide B (NPPB) levels both in the RV (A) and LV (B) of PAB and LAD + PAB rats. Myosin heavy chain 6 (MYH6) was reduced in the LV of LAD rats (C), whereas no significant changes were observed in Myosin heavy chain 7 (MYH7) expression (D). The MYH6/7 ratio was reduced in the LV of LAD rats compared to shams (E), whereas LAD + PAB animals displayed a conserved MYH6/7 ratio. Sarcoplasmic /endoplasmic reticulum Ca2+-ATPase (Serca2A) levels in the LV were similar in all groups (F); however, LAD hearts showed a significant reduction of phosphorylated phospholamban (PLN) levels (G). Data are presented as mean ± standard error of mean and compared using one-way analysis of variance with Tukey post hoc test (A-G). ∗*P* < 0.05. ∗∗*P* < 0.01. ∗∗∗*P* < 0.001. #*P* < 0.05 by Student’s *t*-test. A.U. = arbitrary units; pPLN = phosphorylated phospholamban; TPL = total protein load.

Western blot analysis of lysates from the RV free wall and LV posterior wall (remote from MI area) was performed to evaluate the protein levels of compensatory and hypertrophic markers. Natriuretic peptide B (NPPB) levels were increased in the RV of PAB and LAD + PAB rats (Figure 6A), consistent with RV hypertrophy caused by direct pressure overload.^12^^,^^13^ NPPB levels were also increased in the LV of PAB and LAD + PAB (Figure 6B), indicating that the prohypertrophic signaling in response to PAB is not ventricular-specific and spreads to the LV. LAD rats showed a reduction in Myosin heavy chain 6 (MYH6) levels in the LV (Figure 6C), whereas no significant changes were observed in Myosin heavy chain 7 (MYH7) expression in any group (Figure 6D). These findings resulted in a reduced MYH6/7 ratio in the LV of LAD rats compared to shams (Figure 6E), a shift that is characteristic of end-stage heart failure.^14^, ^15^, ^16^ LAD + PAB animals showed a conserved MYH6/7 ratio, suggesting a more compensated LV phenotype associated with PAB.

LV lysates were analyzed to assess key components of calcium regulation as relating to contractile function. Sarcoplasmic/endoplasmic reticulum Ca2+-ATPase (SERCA2a) levels in the LV were similar in all groups (Figure 6F). However, LAD hearts displayed a significant reduction of the level of the key SERCA2a regulator phosphorylated phospholamban (pPLN/PLN) (Figure 6G), which was otherwise normal in LAD + PAB animals, suggesting a protective role of PAB against LAD-induced dysregulation of calcium handling.

## Discussion

### Development of a small animal model of LV dysfunction treated by PAB

To successfully unmask the hemodynamic and cellular mechanisms activated by PAB, we selected a highly reproducible LAD-ligation rodent model, facilitating the generation of isolated LV dysfunction and testing for the effects of PAB on LV function.^17^ Yerebakan et al^18^ investigated a sheep model of LV failure by selective intracoronary injection of doxorubicin with rescue by PAB. In this model, PAB was shown to induce LV reshaping and improve contractile function, but tissue-level modifications were not specifically evaluated. Because the LV specificity of drug toxicity-based myocardial injury is technically not transferable to small animals, we did not opt for a doxorubicin model. Additionally, the clinical correlation of cancer survivors treated with anthracyclines typically develop biventricular dysfunction^19^ and doxorubicin-treated rats experience increased oxidative stress in the RV and the conduction system.^20^ The potential RV toxicity inherent in the doxorubicin-based LV failure model contraindicates PAB treatment, which requires preserved RV performance (lacks translational relevance).^5^^,^^7^^,^^21^

Macroscopically, the LAD ligation model consistently results in reduced systolic function, eccentric LV dilation, and impaired relaxation as previously reported.^17^^,^^22^^,^^23^ With LAD ligation (alone or before PAB), animals developed LV dilatation and reduced LV systolic and diastolic function early after myocardial injury. In LAD ligation-alone animals, LV dysfunction progressed until experimental termination, confirming progressive LV failure (Figure 2). At a cellular level, LAD ligation activates an acute inflammatory response (days 1-3) characterized by an inflammatory infiltration of neutrophils, macrophages, and monocytes, with accompanying extensive cell death.^24^ The resolving phase (days 7-30) involves tissue digestion, fibroblast activation, and fibrosis.^25^ Our histopathologic analyses documented sustained inflammatory infiltration and extensive fibrosis after LAD ligation. Similar histologic changes have been described in pediatric DCM,^26^ although we recognize that a regional ischemic LV failure model does not perfectly recapitulate the clinical and histologic features of DCM but was chosen for consistency of LV dysfunction induction.

We hypothesized that early controlled RV pressure overload created by PAB provides a therapeutic benefit by inducing immediate positive ventricular-ventricular hemodynamic interactions (reduction of LV dilatation) and through recruitment of healthy remote myocardium to sustain global ventricular contractility. Importantly, we were able to confirm the presence of established LV dysfunction and dilatation between the interval of LAD ligation and PAB treatment, emulating the clinical scenario. Delayed but early treatment of myocardial injury with PAB as implemented in this study recreates the clinical setting in which PAB can be most effective in achieving LV rehabilitation. Clinical series with higher recovery rates after PAB reflect the efficacy of early surgical treatment (few days-weeks after LV failure diagnosis^5^^,^^6^^,^^21^).

### PAB constrains LV dilatation and improves LV systolic and diastolic function

Increasing RV pressure through PAB induces a leftward shift of the interventricular septum and reduces LV end-diastolic dimensions, interrupting the progression of LV eccentric dilatation which was otherwise observed in untreated LAD animals. The favorable reshaping of LV geometry may have promoted the acute improvement in LV systolic function observed in LAD + PAB animals. Similar findings have been documented in patients, where an 11% to 27% increase in LV ejection fraction can be expected in PAB responders.^5^^,^^6^^,^^10^ Children with DCM can develop abnormal septal and LV apical motion, which is associated with electromechanical dyssynchrony and LV systolic dysfunction.^27^ By restoring a more linear interventricular septum, controlled RV pressure overload can counteract this phenomenon. In addition, PAB may optimize LV preload by reducing left atrial volume overload (by partially restricting RV output), which translates into improved LV diastolic function and systo-diastolic performance, as previously described.^10^ The delicate interventricular hemodynamic balance required to optimally achieve LV rehabilitation with RV pressure overload can be modulated in clinical practice by partial de-banding.^10^

Echocardiography revealed that PAB induced a hypertrophic response into the LV posterior wall, which was evident in all animals undergoing RV pressure overload (independently from previous LV injury). We suggest that PAB-mediated prohypertrophic signaling is a paracrine effect that is not ventricular-specific and results in a positive LV adaptive response, which becomes manifest in the presence of LV dysfunction. The source of the beneficial signaling could, at least in part, be due to secreted biomolecules (cytokines, chemokines, growth factors, or other) or cell-cell interactions with infiltrating macrophages (see below).

### PAB induces biventricular adaptive hypertrophy

To determine if selective RV pressure overload can produce adaptive LV hypertrophy, we measured the macroscopic, tissue-level, cellular, and molecular modifications in LV myocardium in response to PAB. Rats treated with PAB displayed increased heart weight, featuring a thicker RV, interventricular septum, and LV posterior wall, as well as cardiomyocyte hypertrophy in both ventricles, compared to LAD and sham animals (Figure 3). A similar increase in LV cardiomyocyte diameter following selective RV pressure overload was documented in rabbits.^28^ The RV and LV are known to respond to pressure overload differently.^29^ RV myocardium preferentially increases transcript and protein expression levels of structural myocardial components and muscle tissue developmental factors,^30^^,^^31^ which support the greater fold change in ventricular mass observed in hearts undergoing PAB vs the mass change seen in the LV in experimental models of transverse aortic constriction.^29^ The molecular signaling activated by RV pressure overload is not limited to RV tissue specifically.^32^^,^^33^ As we observed by western blot analysis of ventricular lysates, PAB triggers increased expression of hypertrophic markers such as NPPB both in the RV and in the LV, in line with our echocardiographic and histologic findings. In addition, proteomic analysis of LV samples of selectively RV-overloaded hearts detected increased expression of adenosine triphosphate synthesis enzymes,^34^ antioxidant defenses, and glycolytic enzymes.^35^ These data suggest that the LV can modify its structural and energetic pathways indirectly as a result of the selective RV overloading. Increased neurohumoral stimulation triggered by the overloaded RV has been hypothesized as mediator of the proteomic changes observed in the not-overloaded LV.^34^ Moreover, altered RV hemodynamic forces (measured by magnetic resonance imaging with 4-dimensional flow) in patients with pulmonary hypertension can modify LV pumping mechanisms,^36^ possibly triggering myocardial fiber stress-related molecular pathways which impact contractility. Yerebakan et al^18^ did not observe LV hypertrophy in their experimental sheep model of doxorubicin-based LV failure treated with PAB. We speculate that the longer duration of “uncontrolled” PAB (RV function was not monitored during their study^18^) is a key differentiating feature from our model, in which we opted for an early termination timepoint to maintain a controlled degree of RV pressure overload (which is achieved by partial de-banding in clinical practice^5^^,^^10^), without inducing RV decompensation and transition to adverse ventricular remodeling. In the early phases of RV pressure overload (2-4 weeks after PAB), Kitahori et al^37^ documented trends of increased LV wall thickness and increased capillary density in the septum (at weeks 2 and 4) and in the LV (at week 2), which parallel our findings. However, after a longer period of uncontrolled RV pressure overload, adverse ventricular remodeling occurs, negatively affecting the RV and the LV.^37^

A key determinant in the transition from compensated to decompensated RV hypertrophy is the maintenance of adequate nutrient flow through angiogenesis to sustain the increased metabolic demands of the hypertrophic myocardium.^38^, ^39^, ^40^, ^41^ Previously, inadequate capillary growth was advanced as a potential pathologic basis for unsuccessful retraining of the morphological LV with PAB in L-transposition of the great arteries.^42^ Decreased RV capillary density in rabbits undergoing PAB triggers RV decompensation that, consequently, also impairs LV performance.^43^ Our histologic analyses show that the biventricular hypertrophy induced by PAB is supported by an increased vessel density in the RV and remote LV, whereas no statistical differences (only a trend) were detected in the peri-infarct region between LAD and LAD + PAB hearts (Figure 4). We speculate that maintaining RV hypertrophy with a compensated phenotype featuring adequate angiogenesis may represent the key aspect for promoting LV rehabilitation. Rats undergoing PAB showed a preserved MYH6/7 ratio, which was otherwise reduced in LAD hearts, further suggesting a compensated hypertrophic response activated by PAB.^14^, ^15^, ^16^

Our model involved a moderate and progressive grade of PAB (allowing the animal to grow into the PAB for a short period), which triggered a compensated biventricular hypertrophic response in the short term. Similarly, in the clinical setting, controlled RV pressure overload is achieved with initially low PAB gradients and close echocardiographic surveillance and partial de-banding during follow-up.^10^^,^^21^ We speculate that this adaptive hypertrophic phenotype may be particularly relevant in the presence of LV dysfunction, where the recruitment of LV myocardial reserve could be crucial to functionally oppose the decline of contractile force and achieve sustainable LV rehabilitation.

### PAB reduces LV myocardial tissue loss after injury

Our histologic findings indicate that PAB limits irreversible myocardial loss and fibrotic replacement after LV injury (Figure 4). LAD + PAB hearts displayed a reduction of LV cellular senescence burden compared to LAD. The induction of fibroblast senescence early after MI has been shown to restrain fibrotic spread, playing a beneficial role in the acute setting.^44^ However, senescent fibroblasts secrete proinflammatory biomolecules which contribute to sustained and expansive tissue injury.^45^^,^^46^ Our model combines acute (LAD ligation) and chronic (PAB) tissue stress, each of which can independently induce cellular senescence. The reduction of LV fibrosis and senescence in concert with an increase in RV fibrosis and senescence, observed in the combined LAD ligation-PAB model, constitutes a novel finding and highlight the independence of RV vs LV responses to specific ischemic and hemodynamic stresses. The precise molecular events which account for modulated changes in LV senescence require further study, although our findings of superimposed senescence and fibrosis histologically suggest that fibroblast senescence may be an unrecognized obligate precursor to pathologic fibrosis. The single timepoint for histologic analysis limits reconstruction of the sequential modifications in cellular senescence during the staggered hemodynamic events and myocardial stressors inherent in LAD + PAB hearts. Increased metabolic and oxidative stress can trigger cardiomyocyte senescence,^47^ which leads to impaired contractility and abnormal conduction.^41^ Consequently, inhibiting cardiomyocyte senescence has been proposed as a novel cardioprotective strategy in ischemic and toxic LV dysfunction.^47^, ^48^, ^49^ We speculate that the compensated hypertrophic response in the LV induced by PAB may reduce the otherwise elevated LV wall stress post MI, including a reduction in oxidative stress, and account for reduced LV senescence and ameliorated LV function.

Dysregulated calcium handling is a common finding in heart failure and is implicated in genetic causes of DCM.^50^, ^51^, ^52^ In the present work, we document how selective RV pressure overload can restore normal calcium cycling in the dysfunctional LV. The SERCA2a is the most important regulator of calcium uptake from the cytosol to the sarcoplasmic reticulum.^50^^,^^51^ SERCA2a activity is regulated by PLN. pPLN promotes increased calcium dynamics via de-repression of PLN-mediated inhibition of SERCA2a function. During the development of heart failure, synergistically decreased SERCA2a levels and dephosphorylation of PLN are typically observed, resulting in reduced calcium reuptake and impaired myocardial contractility.^52^^,^^53^ In line with previous findings indicating that an imbalance of SERCA2a and PLN (even in the presence of preserved SERCA2a expression) results in depressed contractile performance,^52^ we identified a significant increase in dephosphorylated PLN in the LV of LAD hearts. In contrast, LAD + PAB hearts displayed normalized phosphorylation of PLN, suggesting that PAB may improve LV systolic-diastolic function by preserving calcium-mediated cardiac excitation-contraction coupling and sarcoplasmic reticulum calcium stores.

Inflammatory cells may represent a novel cellular paracrine mechanism which can modulate myocardial fibrosis and cellular senescence in the LV in response to PAB.^48^ Acute and chronic RV pressure overload are known to activate a progressive and sequential infiltration of immune cells, mechanistically involved in the process of myocardial healing and/or the reiteration of myocardial injury.^54^^,^^55^ We documented an increased CD68+ macrophage infiltration in the remote LV of LAD + PAB hearts compared to LAD ligation-alone, with equivalent M1/M2 polarization ratio (Figure 5). These findings suggest biological crosstalk between the RV and the LV, in which inflammatory cells might act as cellular mediators to shape distinct ventricular-specific phenotypic responses. We did not observe a bias towards proinflammatory M1 or pro-repair/resolution M2 macrophages between the LAD and LAD + PAB animals, perhaps indicative of the ongoing myocardial stress induced by the PAB or a temporal relationship of benefit for M1 or M2 cells. The beneficial effects of the PAB may result from an overall general increase of macrophages in the myocardium, requiring both M1 and M2 for full effect.

Our work highlights the recommendation that children with DCM treated with PAB undergo proactive clinical surveillance to maintain the adequate grade of RV pressure overload, given that we observed increased macrophage infiltration into the RV with a distinct M1 proinflammatory phenotype, as well as the development of RV fibrosis after PAB. Magnetic resonance imaging in PAB responders documented the presence of de novo RV late gadolinium enhancement positivity late after PAB treatment,^10^ suggesting a state of chronic myocardial stress, the long-term effects of which on RV performance are likely adverse. This combination of our findings and prior clinical observations shows the critical therapeutic index that exists when using PAB to recover LV function.

### Study limitations

The main limitation of a small animal model of LV dysfunction treated by PAB is the impossibility to recapitulate all the features of pediatric DCM in a single mechanism of LV injury, as well as the regional rather than global LV dysfunction created by LAD ligation.^17^ By adopting an ischemic LV failure model, we achieved selective and reproducible LV injury, which features a well-established pathophysiologic phenotype. The comparison of our model with other modes of LV dysfunction treated by PAB will potentially uncover additional reparative cellular and molecular pathways. Future investigations will benefit from cardiac magnetic resonance and cardiac catheterization for a more comprehensive evaluation of LV contractility improvement and LV preload and filling modulation by PAB in larger animal species. The molecular basis of the PAB-induced biventricular hypertrophic response and the biological roles of cellular senescence and inflammation require further in-depth investigations to confirm whether these processes are useful therapeutic targets in their own right in DCM.

## Conclusions

We successfully established a small animal model of LV dysfunction rescued by PAB. Treatment with controlled RV pressure overload induced LV reshaping and ameliorated LV systolic and diastolic dysfunction in the acute and medium term. A compensated hypertrophic phenotype was evidenced by histologic and molecular analysis in both the RV and LV of treated animals, suggesting that PAB may recruit LV myocardial reserve via prohypertrophic signaling to counter the progression of LV dysfunction and promote sustained LV metabolic and functional recovery. The mitigation of irreversible LV myocardial loss and subsequent fibrosis, the preservation of normal calcium regulatory protein levels, as well as the modulation of cellular senescence and inflammatory responses might represent additional explanatory mechanisms induced by PAB that inspire novel treatment approaches in pediatric DCM.

Perspectives **COMPETENCY IN MEDICAL KNOWLEDGE:** PAB can promote LV recovery after injury. Using a small animal model of LV dysfunction treated with PAB, we documented biventricular compensated hypertrophy, which limited the progression of LV dysfunction and promoted sustained LV rehabilitation.

**TRANSLATIONAL OUTLOOK 1:** Controlled RV pressure overload can be adopted to recruit LV functional reserve in infants with DCM and heart failure.

**TRANSLATIONAL OUTLOOK 2:** Future experiments should address the molecular bases of biventricular hypertrophic signaling, as well as the role of cellular senescence and inflammatory response after LV injury.

## Funding Support and Author Disclosures

This work was supported by the Canadian Institutes for Health Research, the Labatt Family Heart Centre Innovation Fund, The Hospital for Sick Children, Toronto, Ontario, Canada; the SickKids Foundation through the Curtis Joseph and Harold Groves Chair in Anesthesia and Pain Medicine and the Department of Anesthesiology and Pain Medicine, University of Toronto, through a Merit Award. Dr Coles has been supported by Coles Professional Medicine Corporation. All other authors have reported that they have no relationships relevant to the contents of this paper to disclose.

## References

[1] Shaddy R.E., George A.T., Jaecklin T. (2018). Systematic literature review on the incidence and prevalence of heart failure in children and adolescents. Pediatr Cardiol.

[2] Weintraub R.G., Semsarian C., Macdonald P. (2017). Dilated cardiomyopathy. Lancet.

[3] Zangwill S. (2017). Five decades of pediatric heart transplantation: challenges overcome, challenges remaining. Curr Opin Cardiol.

[4] Almond C.S., Morales D.L., Blackstone E.H. (2013). Berlin heart EXCOR pediatric ventricular assist device for bridge to heart transplantation in US children. Circulation.

[5] Schranz D., Akintuerk H., Bailey L. (2018). Pulmonary artery banding for functional regeneration of end-stage dilated cardiomyopathy in young children: World Network Report. Circulation.

[6] Spigel Z.A., Razzouk A., Nigro J.J. (2020). Pulmonary artery banding for children with dilated cardiomyopathy: US experience. Semin Thorac Cardiovasc Surg Pediatr Card Surg Annu.

[7] Ponzoni M., Castaldi B., Padalino M.A. (2022). Pulmonary artery banding for dilated cardiomyopathy in children: returning to the bench from bedside. Children (Basel).

[8] Padalino M.A., Crea D., Ponzoni M. (2024). Pulmonary artery banding to treat end-stage heart failure in infants and young children: a multicenter study. JHLT Open.

[9] Latus H., Hachmann P., Gummel K. (2016). Biventricular response to pulmonary artery banding in children with dilated cardiomyopathy. J Heart Lung Transplant.

[10] Ponzoni M., Zanella L., Reffo E. (2023). Late left ventricular myocardial remodeling after pulmonary artery banding for end-stage dilated cardiomyopathy in infants: an imaging study. Int J Cardiol.

[11] Venet M., Malik A., Gold S. (2025). Impact of right ventricular pressure overload on myocardial stiffness assessed by natural wave imaging. JACC Cardiovasc Imaging.

[12] Luo F., Fu M., Wang T. (2023). Down-regulation of the mitochondrial fusion protein Opa1/Mfn2 promotes cardiomyocyte hypertrophy in Su5416/hypoxia-induced pulmonary hypertension rats. Arch Biochem Biophys.

[13] Chen Q.Q., Ma G., Liu J.F. (2021). Neuraminidase 1 is a driver of experimental cardiac hypertrophy. Eur Heart J.

[14] Nakao K., Minobe W., Roden R., Bristow M.R., Leinwand L.A. (1997). Myosin heavy chain gene expression in human heart failure. J Clin Invest.

[15] Gacita A.M., Fullenkamp D.E., Ohiri J. (2021). Genetic variation in enhancers modifies cardiomyopathy gene expression and progression. Circulation.

[16] Abraham W.T., Gilbert E.M., Lowes B.D. (2002). Coordinate changes in Myosin heavy chain isoform gene expression are selectively associated with alterations in dilated cardiomyopathy phenotype. Mol Med.

[17] Ponzoni M., Coles J.G., Maynes J.T. (2023). Rodent models of dilated cardiomyopathy and heart failure for translational investigations and therapeutic discovery. Int J Molec Sci.

[18] Yerebakan C., Boltze J., Elmontaser H. (2019). Effects of pulmonary artery banding in doxorubicin-induced left ventricular cardiomyopathy. J Thorac Cardiovasc Surg.

[19] Boczar K.E., Aseyev O., Sulpher J. (2016). Right heart function deteriorates in breast cancer patients undergoing anthracycline-based chemotherapy. Echo Res Pract.

[20] Rahmanifard M., Vessal M., Noorafshan A., Karbalay-Doust S., Naseh M. (2021). The protective effects of coenzyme Q10 and lisinopril against doxorubicin-induced cardiotoxicity in rats: a stereological and electrocardiogram study. Cardiovasc Toxicol.

[21] Ponzoni M., Frigo A.C., Castaldi B. (2021). Surgical strategies for the management of end-stage heart failure in infants and children: a 15-year experience with a patient-tailored approach. Artif Organs.

[22] Chen J., Chemaly E.R., Liang L.F., LaRocca T.J., Yaniz-Galende E., Hajjar R.J. (2011). A new model of congestive heart failure in rats. Am J Physiol Heart Circ Physiol.

[23] Schnelle M., Catibog N., Zhang M. (2018). Echocardiographic evaluation of diastolic function in mouse models of heart disease. J Mol Cell Cardiol.

[24] Müller-Ehmsen J., Krausgrill B., Burst V. (2006). Effective engraftment but poor mid-term persistence of mononuclear and mesenchymal bone marrow cells in acute and chronic rat myocardial infarction. J Mol Cell Cardiol.

[25] Yang S.M., Liu J., Li C.X. (2014). Intermedin protects against myocardial ischemia-reperfusion injury in hyperlipidemia rats. Genet Mol Res.

[26] Pilati M., Rebonato M., Formigari R., Butera G. (2022). Endomyocardial biopsy in pediatric myocarditis and dilated cardiomyopathy: a tool in search for a role. J Cardiovasc Dev Dis.

[27] Hui W., Slorach C., Friedberg M.K. (2018). Apical transverse motion is associated with interventricular mechanical delay and decreased left ventricular function in children with dilated cardiomyopathy. J Am Soc Echocardiogr.

[28] Apitz C., Honjo O., Humpl T. (2012). Biventricular structural and functional responses to aortic constriction in a rabbit model of chronic right ventricular pressure overload. J Thorac Cardiovasc Surg.

[29] Friehs I., Cowan D.B., Choi Y.-H. (2013). Pressure-overload hypertrophy of the developing heart reveals activation of divergent gene and protein pathways in the left and right ventricular myocardium. Am J Physiol Heart Circ Physiol.

[30] Kreymborg Kg, Uchida S., Gellert P. (2010). Identification of right heart-enriched genes in a murine model of chronic outflow tract obstruction. J Mol Cell Cardiol.

[31] Sheikh A.M., Barrett C., Villamizar N. (2009). Right ventricular hypertrophy with early dysfunction: a proteomics study in a neonatal model. J Thorac Cardiovasc Surg.

[32] Cao Y., Li Y., Wu M. (2020). RNA-sequencing analysis of gene expression in a rat model of acute right heart failure. Pulm Circ.

[33] Roncon-Albuquerque R., Vasconcelos M., Lourenço A.P. (2006). Acute changes of biventricular gene expression in volume and right ventricular pressure overload. Life Sci.

[34] Schott P., Singer S.S., Kögler H. (2005). Pressure overload and neurohumoral activation differentially affect the myocardial proteome. PROTEOMICS.

[35] Faber M.J., Dalinghaus M., Lankhuizen I.M. (2007). Time dependent changes in cytoplasmic proteins of the right ventricle during prolonged pressure overload. J Mol Cell Cardiol.

[36] Pola K., Bergström E., Töger J. (2022). Increased biventricular hemodynamic forces in precapillary pulmonary hypertension. Sci Rep.

[37] Kitahori K., He H., Kawata M. (2009). Development of left ventricular diastolic dysfunction with preservation of ejection fraction during progression of infant right ventricular hypertrophy. Circ Heart Fail.

[38] Frump A.L., Bonnet S., de Jesus Perez V.A., Lahm T. (2018). Emerging role of angiogenesis in adaptive and maladaptive right ventricular remodeling in pulmonary hypertension. Am J Physiol Lung Cell Mol Physiol.

[39] Handoko M.L., de Man F.S., Happé C.M. (2009). Opposite effects of training in rats with stable and progressive pulmonary hypertension. Circulation.

[40] Kolb T.M., Peabody J., Baddoura P. (2015). Right ventricular angiogenesis is an early adaptive response to chronic hypoxia-induced pulmonary hypertension. Microcirculation.

[41] Potus F., Ruffenach G., Dahou A. (2015). Downregulation of microRNA-126 contributes to the failing right ventricle in pulmonary arterial hypertension. Circulation.

[42] Toba S., Sanders S.P., Gauvreau K., Mayer J.E., Carreon C.K. (2022). Histopathologic changes after pulmonary artery banding for retraining of subpulmonary left ventricle. Ann Thorac Surg.

[43] Gold J., Akazawa Y., Sun M., Hunter K.S., Friedberg M.K. (2020). Relation between right ventricular wall stress, fibrosis, and function in right ventricular pressure loading. Am J Physiol Heart Circ Phsyiol.

[44] Zhu F., Li Y., Zhang J. (2013). Senescent cardiac fibroblast is critical for cardiac fibrosis after myocardial infarction. PLoS ONE.

[45] Wang W.-J., Chen X.-M., Cai G.-Y. (2021). Cellular senescence and the senescence-associated secretory phenotype: potential therapeutic targets for renal fibrosis. Exper Gerontol.

[46] Osorio J.M., Espinoza-Pérez C., Rimassa-Taré C. (2023). Senescent cardiac fibroblasts: a key role in cardiac fibrosis. Biochim Biophys Acta Mol Basis Dis.

[47] Huang P., Bai L., Liu L. (2021). Redd1 knockdown prevents doxorubicin-induced cardiac senescence. Aging.

[48] Chen M.S., Lee R.T., Garbern J.C. (2022). Senescence mechanisms and targets in the heart. Cardiovasc Res.

[49] Zheng H., Liang X., Han Q. (2021). Hemin enhances the cardioprotective effects of mesenchymal stem cell-derived exosomes against infarction via amelioration of cardiomyocyte senescence. J Nanobiotechnol.

[50] Zhihao L., Jingyu N., Lan L. (2020). SERCA2a: a key protein in the Ca(2+) cycle of the heart failure. Heart Fail Rev.

[51] Lai P., Nikolaev V.O., De Jong K.A. (2022). Understanding the role of SERCA2a microdomain remodeling in heart failure induced by obesity and type 2 diabetes. J Cardiovasc Dev Dis.

[52] Belke D.D., Swanson E.A., Dillmann W.H. (2004). Decreased sarcoplasmic reticulum activity and contractility in diabetic db/db mouse heart. Diabetes.

[53] Schmidt A.G., Edes I., Kranias E.G. (2001). Phospholamban: a promising therapeutic target in heart failure?. Cardiovasc Drugs Ther.

[54] Watts J.A., Gellar M.A., Obraztsova M., Kline J.A., Zagorski J. (2008). Role of inflammation in right ventricular damage and repair following experimental pulmonary embolism in rats. Int J Exper Pathol.

[55] Yoshida K., Abe K., Ishikawa M. (2019). Inhibition of TLR9-NF-κB–mediated sterile inflammation improves pressure overload-induced right ventricular dysfunction in rats. Cardiovasc Res.

