# Supplementary material for: Controlled Right Ventricular Pressure Overload Can Rescue Left Ventricular Dysfunction by Promoting Biventricular Adaptive Hypertrophy
Source: JACC Basic Transl Sci. 2025 Jun 18;10(8):101259. doi: 10.1016/j.jacbts.2025.02.018 (PMC12399129; doi:10.1016/j.jacbts.2025.02.018)
Supplement: Supplemental Material [file mmc1.docx]

**Supplemental Appendix**

Supplemental methods: page 2

Supplemental results: page 7

Supplemental figure legends: page 9

Supplemental references: page 19

**Supplemental Methods**

*LV dysfunction model*

After anesthesia induction with 4% isoflurane, rats underwent oral intubation and mechanical ventilation. Anesthesia was maintained using 2% isoflurane driven by 100% oxygen (2 L/min); heart rate, and arterial oxygen saturation were monitored continuously using a pulse oximeter, and body temperature using a rectal probe and heating pad. A left anterior thoracotomy in the fourth intercostal space was performed, the pericardium was opened, and the LAD was identified. Using a 7-0 polypropylene suture the LAD was permanently ligated approximately 5 mm below the left atrial appendage^1^. Blanching of the LV anterior surface and apex confirmed successful LAD ligation in all cases.

*PAB model*

Animal preparation and anesthesia were conducted as above. A left anterior thoracotomy in the third intercostal space was performed, the pericardium was opened, and the pulmonary artery mobilized. A small surgical clip (LT100 ETHICON) was half-closed around the main pulmonary artery using a clip applier (LX107 ETHICON) with a stopper to calibrate an inner diameter of the clip corresponding to 18G^2, 3^.

*LV dysfunction model rescued by PAB*

Animals underwent permanent LAD ligation as described above. One week later and after echocardiographic confirmation of LV systolic dysfunction and dilatation, the rats underwent PAB using the same surgical protocol as above.

*Echocardiography*

After induction with 4% isoflurane, anesthesia was maintained using 2% isoflurane driven by 100% oxygen (2 L/min) via nose cone. Echocardiography was performed using a Vivid-E9 ultrasound system with a 12-MHz phased array transducer (GE, Horten, Norway) and electrocardiographic monitoring by a single blinded operator. Raw data were stored for offline analysis on commercially available software (EchoPAC version 201; GE, Horton, Norway). M-mode and two-dimensional short-axis, parasternal, and apical 4-chamber images were acquired to measure biventricular dimensions and systolic function (expressed as fractional area change, ejection fraction, and tricuspid annular plane systolic excursion [TAPSE; placing the M-mode cursor through the tricuspid annulus at the RV free wall]). Two-dimensional short-axis view at the aortic valve level was used to measure RV and LV outflow velocities and gradients by CW Doppler. Diastolic function was assessed using mitral valve (MV) early (E) and late (A) peak inflow velocities by PW Doppler with the sample volume placed at the tip of MV and tricuspid valve leaflets and parallel to the blood flow in the apical 4-chamber view^4–6^. Isovolumic contraction time (IVCT), ejection time (ET), and isovolumic relaxation time (IVRT) were measured from PW Doppler flow across the pulmonary-tricuspid valves with simultaneous ECG monitoring in the apical 4-chamber view. Myocardial performance index was calculated as (IVCT + IVRT)/ET^4–6^.

*Histological analysis*

After 4 weeks from initial surgery, the heart was harvested under deep anesthesia, washed with saline, dry weighted, and sent for further processing.

*Hematoxylin-eosin staining and hypertrophy analysis*

Rat heart was dissected and fixed with 10% formalin overnight, then dehydrated in an ethanol series, cleared with xylene, and embedded in paraffin. To evaluate the ventricular thickness, the paraffin embedded rat heart was sectioned at 7μm thickness using a microtome. The sections were stained using Hematoxylin-eosin Stain Kit (Abcam Cat# 245880) as directed by the manufacturer. The sections were scanned using 3DHistech Slide Scanner with CaseViewer software. The images were analyzed by an observer blinded to the treatment group, and the ventricular thickness was measured at the RV free wall, interventricular septum, and LV posterior wall using ImageJ. To account for differences in animals’ body size, ventricular wall thickness was indexed to the animal’s body weight (μm/g). Cardiomyocyte diameter was measured at level of the nucleus in longitudinal sections of myocardial fibers in the RV free wall and LV posterior and lateral wall (the interventricular septum was excluded to avoid overestimation resulting from PAB-induced afterload effects), as previously described^7, 8^.

*Trichrome staining and fibrosis analysis*

Paraffin-embedded heart tissue was sectioned at 7μm thickness using a microtome. The sections were stained using Trichrome Stain Kit (Abcam Cat# 150686) as directed by the manufacturer. The sections were scanned using 3DHistech Slide Scanner with CaseViewer software. The images were analyzed by an observer blinded to the treatment group, and the % fibrosis area to total area were measured using ImageJ.

*Senescence associated β-Galactosidase staining and analysis*

Rat heart was dissected and fixed with 10% formalin overnight and the hearts were embedded in OCT and stored at -20°C. The hearts were sectioned at 10μm thickness using cryostat and stored at -20°C. The sections were stained for senescence-associated β-galactosidase using senescence detection kit (Abcam Cat# 65351) as directed by the manufacturer. The sections were scanned using 3DHistech Slide Scanner with CaseViewer software. The images were analyzed by an observer blinded to the treatment group and the % beta-galactosidase positive area to total area was measured using ImageJ.

*Immunofluorescence, angiogenesis, and inflammatory infiltration analysis*

The heart sections were rehydrated and antigen retrieval was conducted using Antigen retrieval solution (Sigma Aldrich Cat# C9999). The sections were permeabilized with 0.5% Triton X-100 in PBS and, after blocking with 3% BSA, were incubated overnight at 4°C in a moist chamber with the primary antibody. After four washes with PBS, sections were incubated with secondary antibodies diluted 1:400 in PBS containing 1% BSA. After 3 consecutive washes with PBS, nuclear stain Hoechst 33342 (1:10000 in PBS, Sigma Aldrich Cat# 40046) was applied for 5 minutes and followed by 2 consecutive 10-min washes. The coverslips were subsequently mounted (Dako Cat# S3022) and allowed to dry overnight. Negative controls lacking primary antibody were processed similarly. The sections were viewed and photographed using a Zeiss LSM880 Airyscan microscope. Zen black software was used to process the captured images. The primary antibodies used were mouse anti-CD68 (1:100, Abcam), rabbit anti-CD206 (1:200, Abcam), rabbit anti-iNOS (1:100, Novus Biotech), α-smooth muscle actin (α-SMA) (1:400, Abcam) and Von Willebrand Factor (VWF) (1:100, Abcam). The number of vessels in the RV and LV (distinguished in remote LV and peri-MI region) was counted and indexed to the myocardial area. Ten random high-power-field (HPF) images were taken from the RV, remote LV, and peri-MI region of each animal and the number of CD68+ (total macrophages), CD68/iNOS+ (M1 macrophages), and CD68/CD206+ (M2 macrophages) cells were counted and averaged. All analyses were performed by an observer blinded to the treatment group.

*Western blot analysis*

Myocardial tissue from the RV free wall and LV posterior wall (remote from MI area) was homogenized in RIPA buffer with Protease Inhibitor Cocktail (Roche, ON, Canada) and Pierce Phosphatase Inhibitors added (Thermo Fisher Scientific) using 2 cycles of agitation (2 min at 20 Hz) of TissueLyser II (Qiagen). Homogenates were centrifuged (20 mins at 15,000xg) and protein content of the supernatants were quantified using Pierce BCA Protein Assay Kit (Thermo Fisher Scientific). Western blot samples were prepared by addition of Laemmli loading buffer (2% SDS, 0.0005% bromophenol blue, 10% glycerol, 63 mM Tris-HCl and 0.1% 2-mercaptoethanol) to supernatants and heating for 10 min at 75°C. Samples were loaded and separated on 4–20% (w/v) gradient SDS-PAGE gels (GenScript) and transferred to 0.2 μm PVDF membranes with a Trans-Blot Turbo Transfer System (10 mins at 2.5A and 25V) (Bio-Rad, CA, USA). Membranes were blocked (5% blocking solution in TBS) for 1 hour, followed by overnight primary antibody incubation at 4 °C (5% blocking solution in TBS-T). Following 15 min wash in TBS-T, membranes were incubated in species specific horseradish peroxidase (HRP)-linked secondary antibody (5% blocking solution in TBS-T) at room temperature for 1 hr. Membranes were washed for 15 min wash in TBS-T and protein levels were measured by imaging on a Li-Cor Odyssey with SuperSignal West Femto Maximum Sensitivity Substrate (Thermo Fisher Scientific). Total protein loading was measured using ponceau stain (10 min with agitation followed by 5 min wash in water). ImageJ was used to obtain optical densities and protein content was normalized to total protein loading (National Institutes of Health, https://imagej.nih.gov). All samples derived from same experiment and gels/blots were processed in parallel. The following primary antibodies were used (abbreviation; company, catalogue number, concentration): natriuretic peptide type B (NPPB; Invitrogen, PA5-96084, 1:1000), phospholamban (PLN; Invitrogen, PA5-119803, 1:1000), phosphoPLN (Ser16, Thr17) (pPLN; Invitrogen, PA5-114620, 1:1000), sarcoplasmic reticulum calcium ATPase 2 (Serca2A; Abcam, 150435, 1:100,000), myosin heavy chain 6 (MYH6; Santa Cruz, 168676, 1:200) and myosin heavy chain 7 (MYH7; Invitrogen, PA5-110000, 1:1000). The following secondary antibodies were used (company, catalogue number, concentration): goat anti-rabbit IgG HRP (BioRad, 170615, 1:3000) and rabbit anti-goat IgG (Abcam, 97100, 1:5000).

**Supplemental Results**

*Survival*

A total of 12 rats died before the experimental termination. In the SHAM group, 2 rats died during sham surgery. In the PAB group, 1 rat died during PAB surgery and 1 died 3 weeks after PAB. In the LAD group, 2 rats died during LAD ligation procedure and 1 rat 3 days after LAD. In the LAD+PAB group, 1 rat died during LAD ligation procedure, 1 post-LAD ligation (before PAB), 1 during PAB procedure (due to bleeding), and 2 in the first week after PAB.

*Echocardiographic results*

At baseline, rats presented similar biventricular dimensions, systolic and diastolic function, and normal pressure gradients across the pulmonary artery (data not shown). Early after LAD ligation (week 1), LV dilatation occurred: LV end-diastolic area and LV end-diastolic volume significantly increased in the LAD and LAD+PAB groups, compared to SHAM and PAB (mean ±SEM LV end-diastolic area: LAD 0.55±0.02 cm^2^, LAD+PAB 0.53±0.02 cm^2^, SHAM 0.44±0.02 cm^2^, PAB 0.37±0.02 cm^2^, p<0.001, **Supplemental** **Figure 1A**; mean ±SEM LV end-diastolic volume: LAD 0.55±0.04 mL, LAD+PAB 0.54 ±0.03 mL, SHAM 0.44±0.02 mL, PAB 0.31±0.02 mL, p<0.001, **Supplemental Figure 1B**). Concurrently, LV contractile function was impaired (mean ±SEM LV fractional area change: LAD 0.32±0.02, LAD+PAB 0.34±0.04, SHAM 0.54±0.01, PAB 0.51±0.02, p<0.001, **Supplemental Figure 1C**; mean ±SEM LV ejection fraction: LAD 0.35±0.01, LAD+PAB 0.38±0.01, SHAM 0.53±0.01, PAB 0.53±0.01, p<0.001, **Supplemental Figure** **1D** and **Supplemental Figure 2**) and diastolic function deteriorated (mean ±SEM MV inflow Doppler E/A: LAD 1.97±0.10, LAD+PAB 1.91±0.08, SHAM 1.33±0.07, PAB 1.45±0.14, p<0.001, **Supplemental Figure 1E**; mean ±SEM LV myocardial performance index: LAD 0.80±0.03, LAD+PAB 0.81±0.02, SHAM 0.62±0.02, PAB 0.82±0.03, p<0.001, **Supplemental Figure 1F**). Right ventricular dimensions and systolic and diastolic function remained unaltered (**Supplemental Figure 3**). Pressure gradients across the PAB and the LV outflow tract are reported in **Supplemental Figure 4**. PAB induced a progressive increase in RV pressure overload in the PAB and LAD+PAB groups. None of the animals developed echocardiographic signs of LV outflow tract obstruction.

**Supplemental Figures**

**Supplemental Figure 1.** Echocardiographic findings at week 1. LAD and LAD+PAB animals developed LV dilatation compared to SHAM and PAB rats, as measured by increased LV end-diastolic area (A) and end-diastolic volume (B). Concurrently, LV fractional area change (C) and LV ejection fraction (D) decreased in LAD and LAD+PAB, while MV inflow Doppler E/A (E) and LV myocardial performance index (F) increased, indicative of LV systolic and diastolic dysfunction. Panel G and H show echocardiographic 4 chamber views during end-diastolic (G) and end-systolic (H) phases: note LV dilatation in LAD and LAD+PAB groups, together with impaired contractile function. Panel I depicts examples of MV inflow Doppler waves: LAD and LAD+PAB present an increased E/A ratio. Data are presented as mean ±SEM and compared using one-way ANOVA with Tukey post-hoc test (panel A, B, C, D, E, F). *p<0.05, **p<0.01 and ***p<0.001. LV: left ventricle. MV: mitral valve.


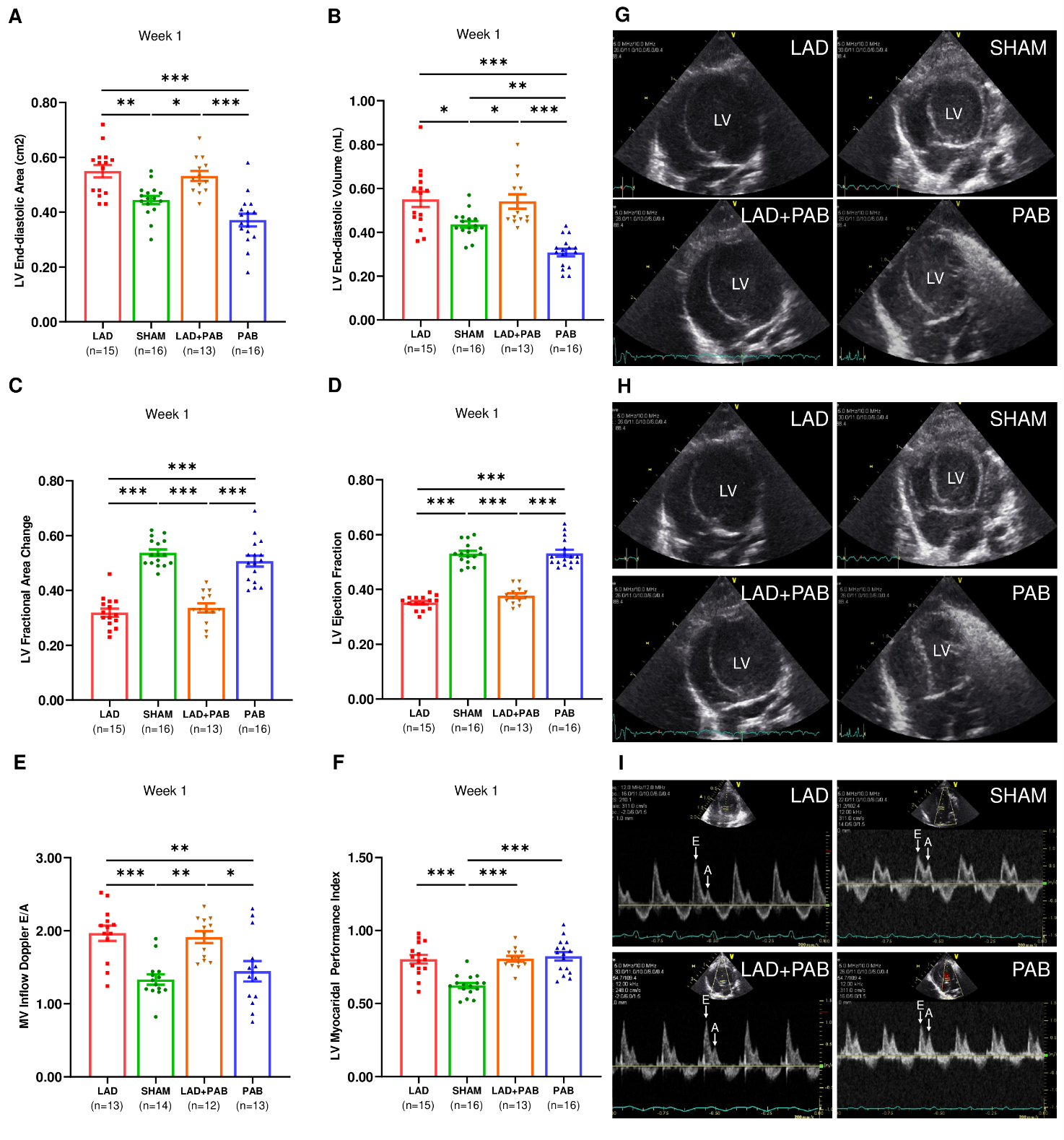


**Supplemental Figure 2.** Echocardiographic evaluation of LV end-diastolic volume (A) and LV ejection fraction (B) at each time point. In panel A, note LV dilatation in LAD and LAD+PAB groups at week 1. While LV dilatation progresses in LAD animals until week 4, after PAB treatment LAD+PAB animals normalize LV dimensions. Panel B shows reduced LV ejection fraction at week 1 in LAD and LAD+PAB animals. Left ventricular systolic dysfunction persists until week 4 in LAD animals, but it is ameliorated by PAB in LAD+PAB animals. Data are presented as mean ±SEM and compared using one-way ANOVA with Tukey post-hoc test performed at each time point (panel A, B). *p<0.05, **p<0.01 and ***p<0.001. LV: left ventricle.


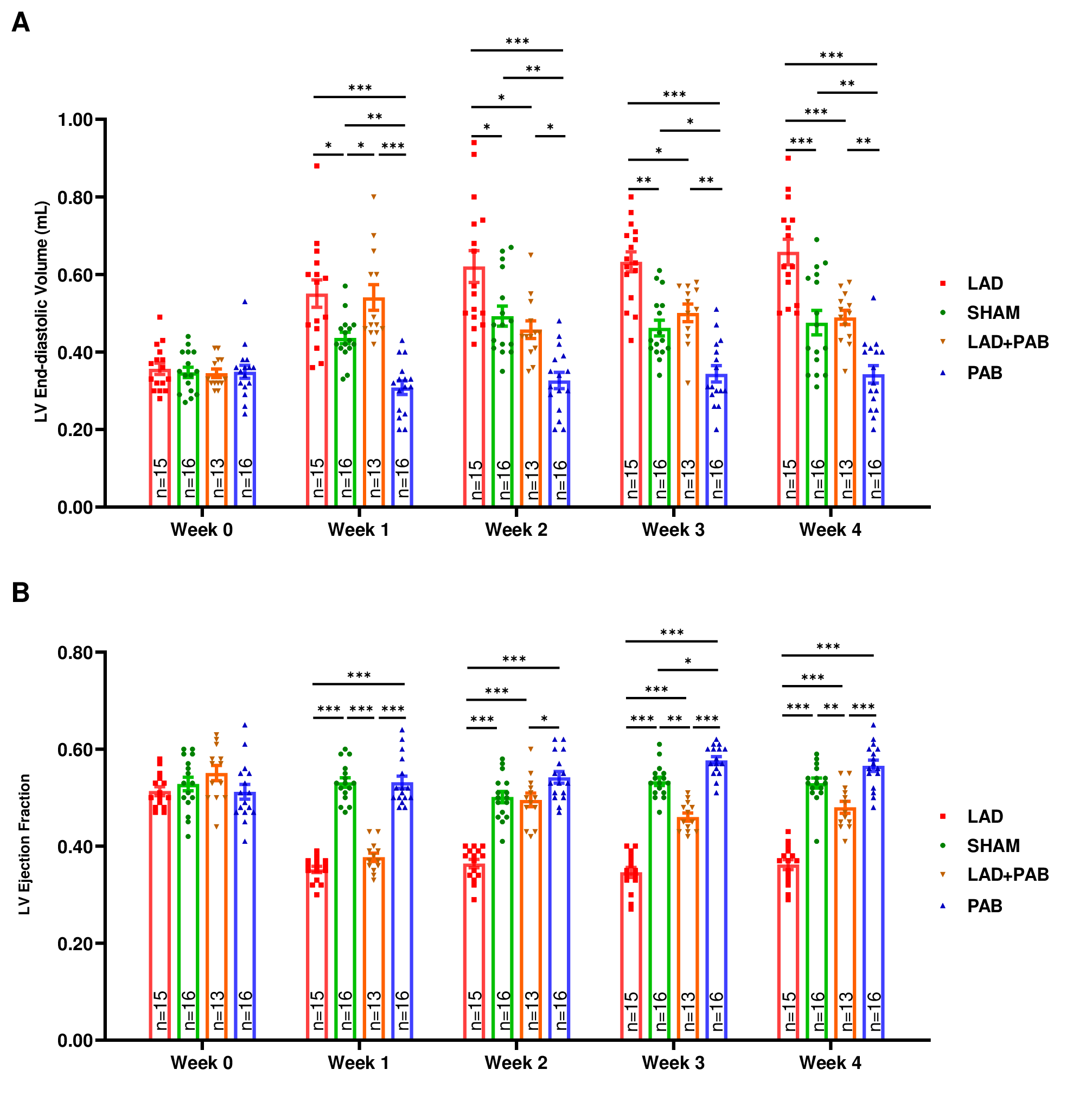


**Supplemental Figure 3.** Echocardiographic evaluation of RV dimensions, RV systolic and diastolic function, and RV and LV thickness at week 1 (panel A, C, E, G, I) and week 4 (panel B, D, F, H, J). RV end-diastolic area is similar among all study groups at week 1 (A), but at week 4, LAD+PAB and PAB animals develop RV dilatation (B). At week 1, RV fractional area change is decreased in PAB animals only (C), while it is reduced in LAD, LAD+PAB, and PAB animals, compared to shams at week 4 (D). Right ventricular diastolic function (measured by TV inflow Doppler E/A) is similar among groups at week 1 (E) and week 4 (F). At week 1, RV hypertrophy is present only in PAB animals (G), while it is evident in LAD+PAB and PAB rats at week 4 (H). Similarly, LV hypertrophy is noticed in PAB animals at week 1 (I) and in PAB and LAD+PAB animals at week 4 (J). Panels K and L depict examples of echocardiographic 4 chamber views (K) and short axis views (L) during end-diastole at week 1 and week 4: note RV dilatation and hypertrophy, together with flattening of the interventricular septum, in LAD+PAB and PAB animals at week 4. Data are presented as mean ±SEM and compared using one-way ANOVA with Tukey post-hoc test (panel A, B, C, D, E, F, G, H). *p<0.05, **p<0.01 and ***p<0.001. LV: left ventricle. RV: right ventricle. TV: tricuspid valve.


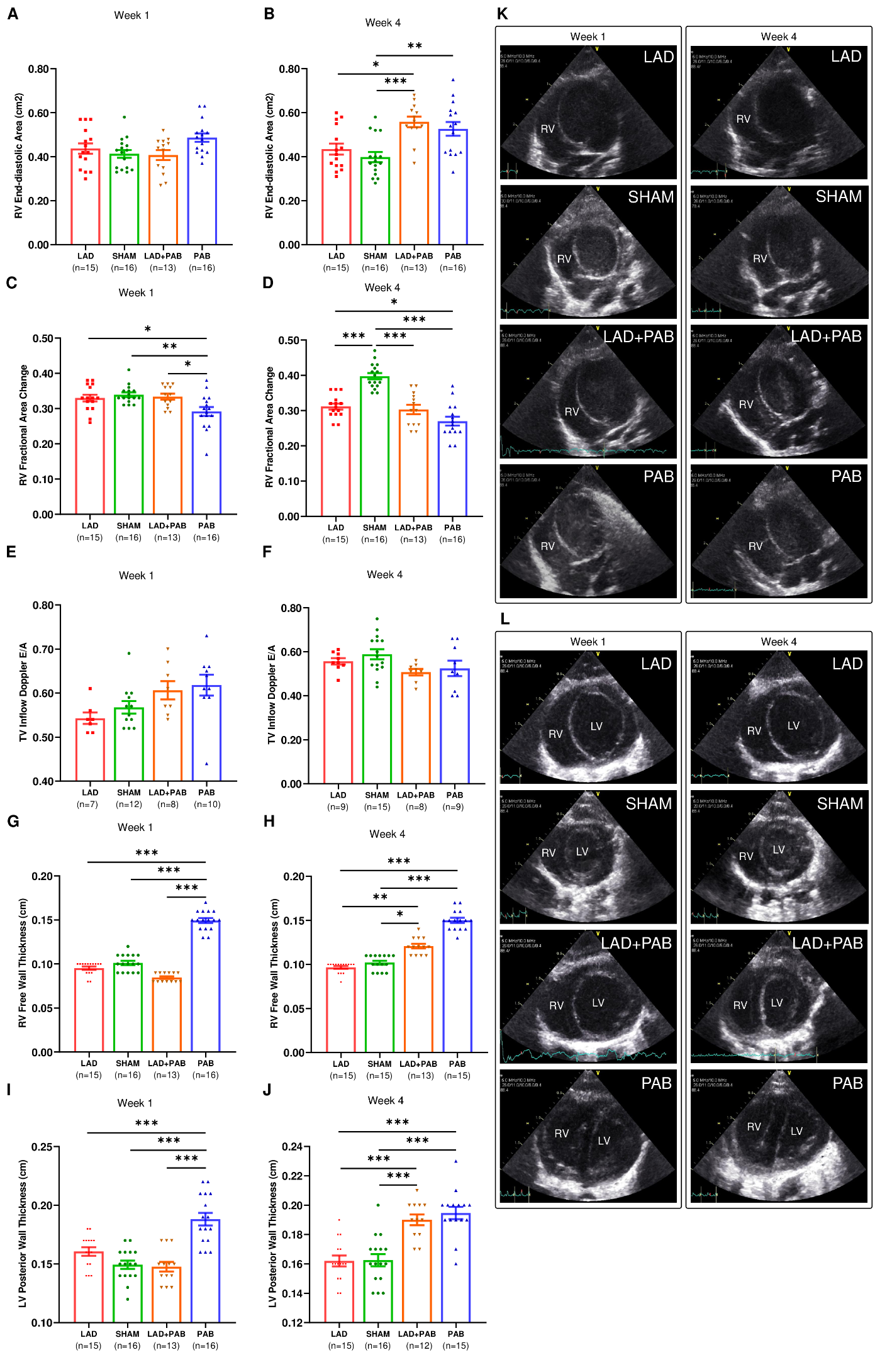


**Supplemental Figure 4.** Echocardiographic evaluation of peak pressure gradients across PAB using CW Doppler at week 1 (panel A) and week 4 (panel B and C), showing significant RV pressure overload in PAB and LAD+PAB rats. Echocardiographic evaluation of peak pressure gradients across LV outflow tract using CW Doppler at week 1 (panel D) and week 4 (panel E and F), demonstrating the absence of LV outflow tract obstruction. Data are presented as mean ±SEM and compared using one-way ANOVA with Tukey post-hoc test (panel A, B, C, D). *p<0.05, **p<0.01 and ***p<0.001.


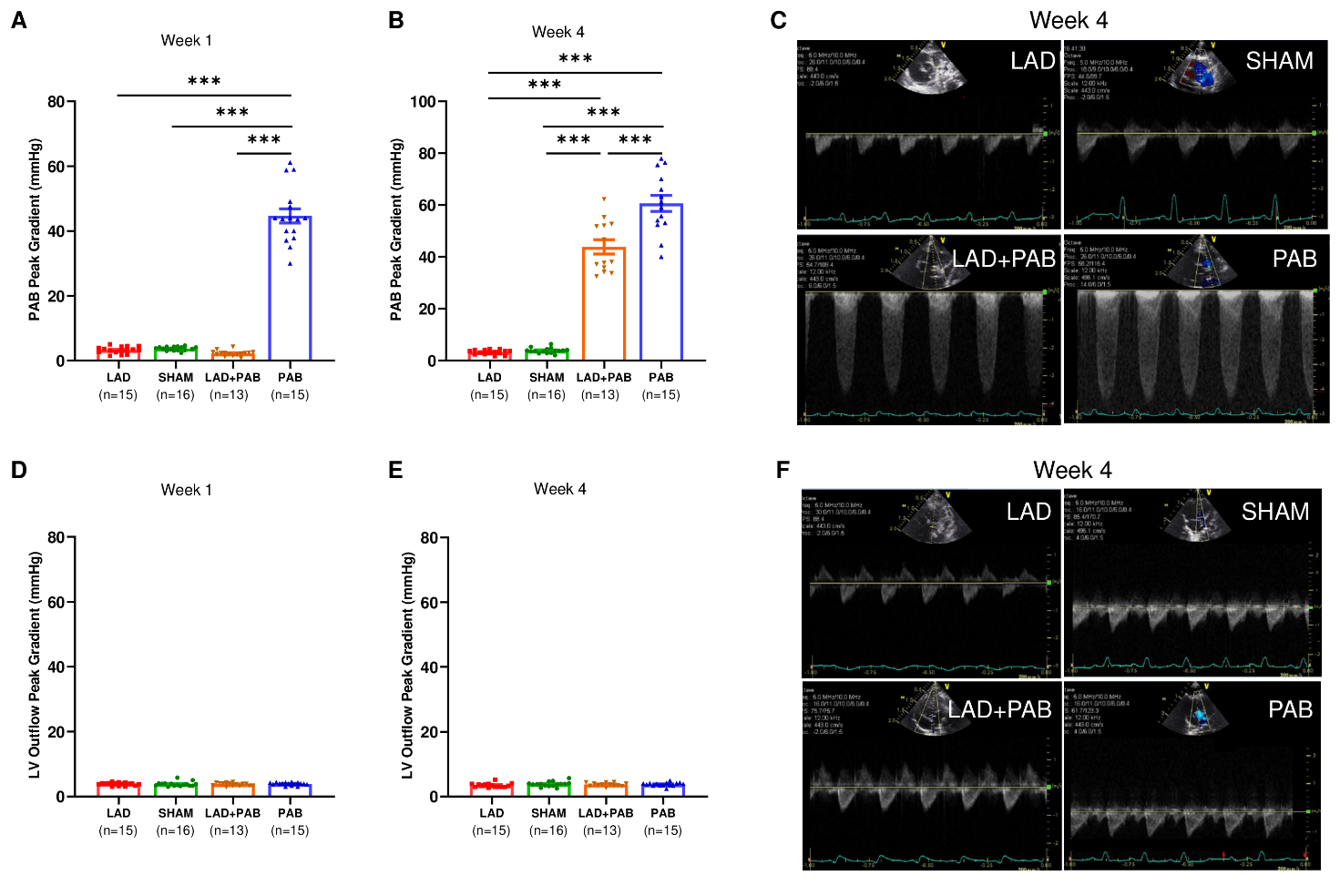


**Supplemental Figure 5.** Examples of high-power fields (105x magnification) of CD68 (red), CD206 (green), and DAPI (blue) immunofluorescence staining of remote LV and peri-MI tissue: white arrows indicate CD68 and CD206 positive cells (M2 macrophages). LV: left ventricle. MI: myocardial infarction.


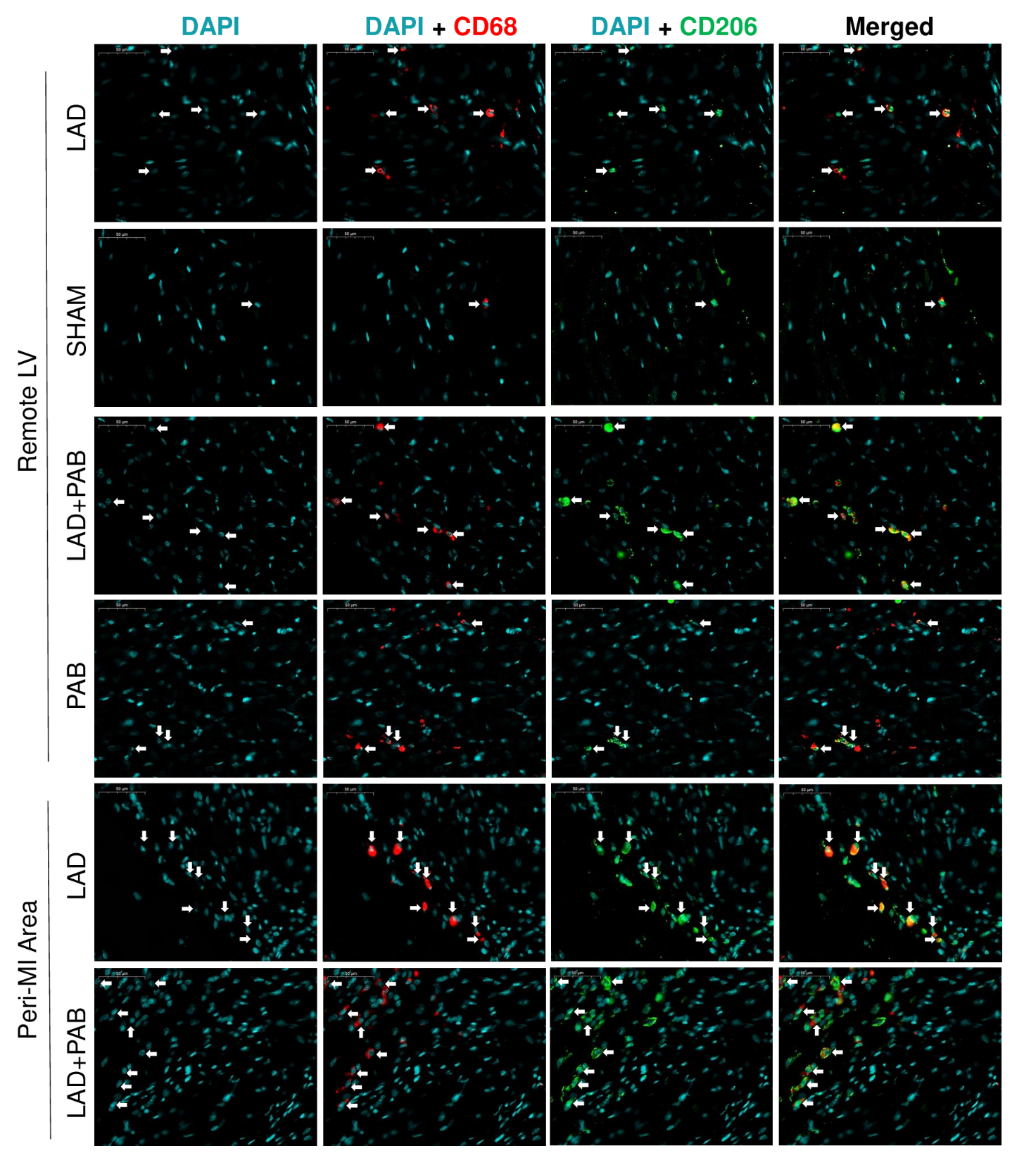


**Supplemental Figure 6.** Results of immunofluorescent staining for macrophages markers in the RV. Total number of CD68 positive cells (macrophages) per HPF in the RV was higher in LAD+PAB and PAB hearts compared to SHAM (A). M1/M2 ratio was increased in LAD+PAB and PAB hearts compared to SHAM and LAD (B), suggesting a pro-inflammatory activation of macrophages in response to PAB-related chronic stress. Panel C shows examples of high-power fields (105x magnification) of CD68 (red), iNOS (green), and DAPI (blue) immunofluorescence staining of RV myocardial tissue: white arrows indicate CD68 and iNOS positive cells (M1 macrophages). Panel D shows examples of high-power fields (105x magnification) of CD68 (red), CD206 (green), and DAPI (blue) immunofluorescence staining of RV myocardial tissue: white arrows indicate CD68 and CD206 positive cells (M2 macrophages). Data are presented as mean ±SEM and compared using one-way ANOVA with Tukey post-hoc test (panel B) or as median (Q1-Q3) and compared with Kruskal-Wallis test with Dunn's post-hoc test (panel A). *p<0.05, **p<0.01 and ***p<0.001. HPF: high power field. LV: left ventricle. MI: myocardial infarction. RV: right ventricle.


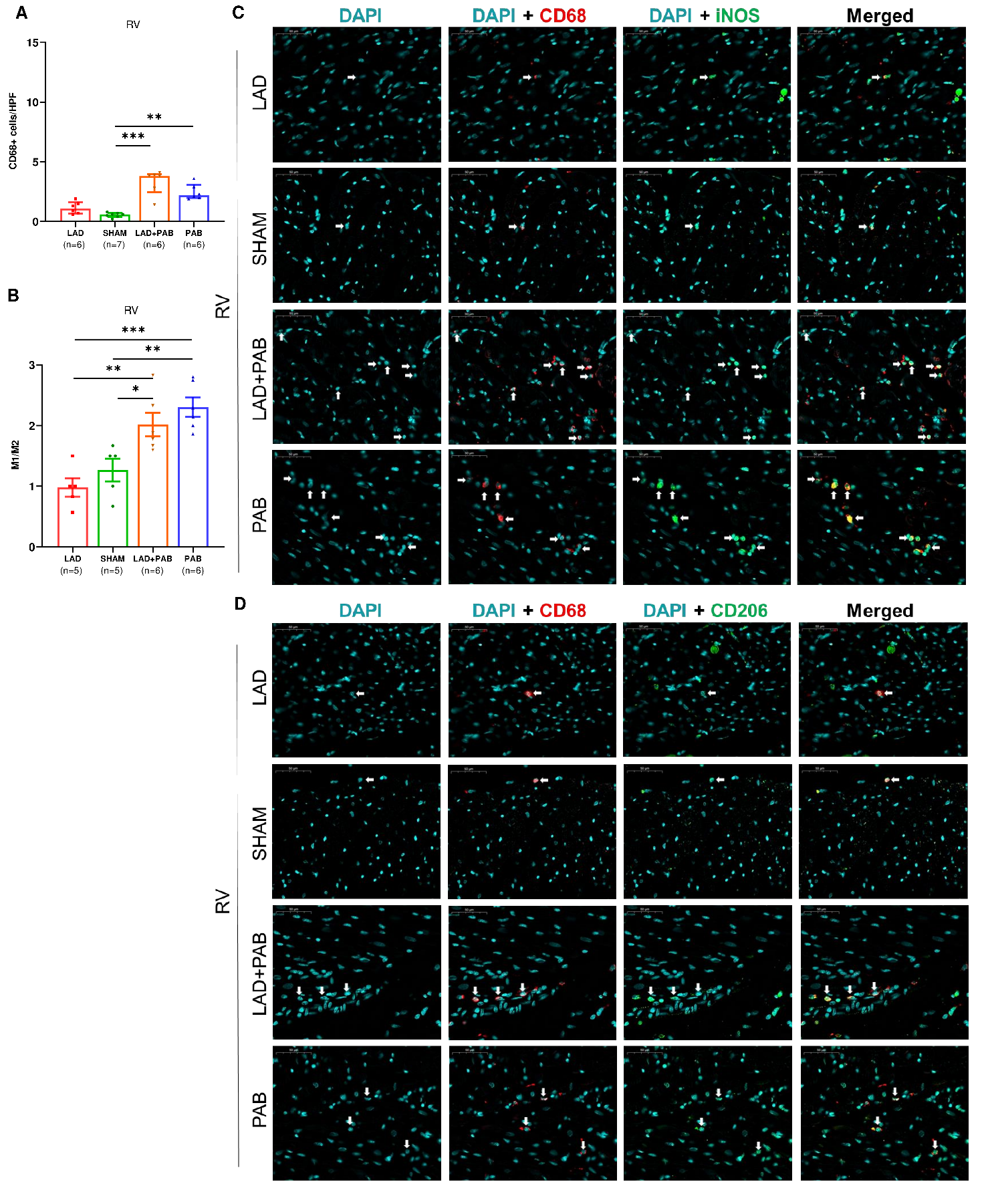


**Supplementary References**

1. Kolk MV V, Meyberg D, Deuse T, et al. LAD-ligation: a murine model of myocardial infarction. *J Vis Exp*. 2009. Published onlineOctober 2009. https://doi.org/10.3791/1438.

2. Hirata M, Ousaka D, Arai S, et al. Novel Model of Pulmonary Artery Banding Leading to Right Heart Failure in Rats. *Biomed Res Int*. 2015;2015:753210.

3. Ponzoni M, Coles JG, Maynes JT. Rodent Models of Dilated Cardiomyopathy and Heart Failure for Translational Investigations and Therapeutic Discovery. *Int J Mol Sci*. 2023;24:3162.

4. Zacchigna S, Paldino A, Falcão-Pires I, et al. Towards standardization of echocardiography for the evaluation of left ventricular function in adult rodents: a position paper of the ESC Working Group on Myocardial Function. *Cardiovasc Res*. 2021;117:43–59.

5. Rosas PC, Neves LAA, Senese PB, Gralinski MR. Comprehensive Echocardiographic Assessment of Right Ventricle Function in a Rat Model of Pulmonary Arterial Hypertension. *J Vis Exp*. 2023. Published onlineJanuary 20, 2023. https://doi.org/10.3791/63775.

6. Schnelle M, Catibog N, Zhang M, et al. Echocardiographic evaluation of diastolic function in mouse models of heart disease. *J Mol Cell Cardiol*. 2018;114:20–28.

7. Baudouy D, Michiels J-F, Vukolic A, Wagner K-D, Wagner N. Echocardiographic and Histological Examination of Cardiac Morphology in the Mouse. *J Vis Exp*. 2017. Published onlineOctober 26, 2017. https://doi.org/10.3791/55843.

8. Huang J, Zhang W, Zhang C, Wang L. Interleukin-17 aggravates right ventricular remodeling via activating STAT3 under both normoxia and hypoxia. *BMC Cardiovasc Disord*. 2021;21:249.
